# An Evaluation of the Arginine Requirements of Broiler Chickens and the Potential Arginine and Energy-Saving Effects of Guanidinoacetic Acid †

**DOI:** 10.3390/ani15010004

**Published:** 2024-12-24

**Authors:** Adriaan Verhelle, Behnam Saremi

**Affiliations:** 1 Poulpharm BV, Prins-Albertlaan 112, 8870 Izegem, Belgium; adriaan.verhelle@poulpharm.be; 2 CJ Europe GmbH, Amino Acid Application Center, Unterschweinstiege 2-14, 60549 Frankfurt am Main, Germany

**Keywords:** broiler chicken, L-arginine, guanidinoacetic acid, nutrition, amino acids

## Abstract

Two 35-day trials were conducted to determine the arginine (Arg) requirement of broiler chickens and the Arg and energy-sparing effects of guanidinoacetic acid (GAA). In experiment 1, a low-Arg diet (basal diet) was supplemented with increasing levels (0.06–0.61%) of L-Arg or GAA. In experiment 2, a diet meeting the energy and amino acid requirements of broiler chickens served as the positive control (PC). Two negative control (NC) groups were assigned by reducing either 50 (NC1) or 100 (NC2) kcal nitrogen-corrected metabolizable energy. Test groups were supplemented with 0.06% GAA for NC1 and 0.12% GAA for NC2 to compensate for the lower energy in the feed. The low dietary Arg concentration (starter: 1.02%, grower: 0.88%, finisher: 0.75%) significantly reduced overall performance (*p* < 0.05). Supplementation of either L-Arg or GAA with a low-Arg diet both alleviated the lower performance (*p* < 0.05). However, more GAA was needed to provide the same level of growth attained with L-Arg at an equivalency rate of 1 GAA to 0.57 Arg. Reduction of dietary energy by 50 and 100 kcal did not significantly influence the performance of birds at all stages of growth. Moreover, no effect of GAA supplementation at 0.06% or 0.12% was observed.

## Simple Summary

Poultry are unable to synthesize arginine. Thus, it is essential to include enough arginine in poultry feed. One can save some arginine by means of providing the molecules in which arginine is needed as a building block. However, the question is how much arginine can these kinds of molecules replace in poultry feed. Herein, these questions are addressed in two trials comparing guanidinoacetic acid and arginine. Moreover, the energy-saving potential of guanidinoacetic acid is evaluated. Optimal performance in broiler chickens was achieved when birds were provided with higher dietary arginine content compared to the available recommendation tables. Supplementation of either arginine or guanidinoacetic acid with a low-arginine feed alleviated the lower performance. However, more guanidinoacetic acid was needed to provide the same level of growth attained with arginine (1 to 0.57 ratio). Reduction of dietary energy by 50 and 100 kcal/kg did not significantly influence the performance of birds at all stages of growth. Moreover, guanidinoacetic acid had no energy-sparing effect. This study helps the industry to accurately meet the arginine and energy requirements of broiler chickens.

## 1. Introduction

Feed is a major cost of meat production [1]. In a typical poultry diet, energy, protein (amino acids: AA), and phosphorus are the most expensive. Feed additives that can spare energy and/or AA are of great interest, especially during times when the feed raw materials are scarce and expensive.

Birds have both a high Arg requirement and an incomplete urea cycle [2]. Due to this, birds cannot synthesize arginine (Arg). The importance of Arg in poultry species is further accentuated in the fact that together with glycine (Gly), Arg is required in the synthesis of creatine (Cre) via the intermediate guanidinoacetic acid (GAA) [3,4]. The addition of Arg to an Arg-deficient diet increased the Cre content in broilers’ tissues [5], thus supporting the energy homeostasis in muscle under physiologically controlled conditions. As Cre levels reach physiological optimal levels, Cre acts as a negative feedback signal to Arg:Gly aminotransferase (AGAT), the enzyme responsible for the formation of GAA out of Gly and Arg [6,7,8]. Consequently, there is no more Arg and methionine (Met) being drained for unnecessary synthesis of GAA and Cre [9].

Arg, as a functional amino acid, also plays an important role during protein synthesis for growth, improvement of health, and reproduction [10]. Arg further serves as a precursor for nitric oxide, which acts as a vasodilator in the regulation of blood flow [11]. This function is particularly significant to birds challenged with pulmonary hypertension when reared at high altitudes or undergoing heat stress. Additionally, Arg is involved in hormone production and release [12] and plays a key role in immunity [13,14,15], as cited by [11].

In the context of poultry nutrition, Arg is identified as the fifth amino acid that becomes limiting in the synthesis of proteins or growth when dietary intake falls short. Arg requirements vary between 1.08 and 1.25 standardized ileal digestible (SID) Arg to lysine (Lys) [16,17,18]. Among the feed ingredients, animal proteins are good sources of Arg. Currently, the availability and cost of these protein sources limit their usage in broiler feed formulations. However, the advent of fermentation-driven L-Arg production makes it convenient to easily meet the dietary Arg requirements (no upper or lower limitations of use; [19]).

GAA is an intermediary metabolite produced endogenously from Arg and Gly in the creatine pathway [4]. Although non-proteogenic in itself, methylation using methionine (Met) produces Cre, an important compound that carries and stores phosphate for ATP production [4]. Extra GAA will be excreted in urine at 0 to 6.175 and 99.25 mg/kg metabolic weight per day in diets supplemented with 0, 0.6, and 6 g/kg GAA [20]. Cre is mainly involved in the energy metabolism of muscle, heart, brain, sperm, and immune cells [21]. The level of Cre in the body depends on the availability of certain AAs, among others Arg [22].

Chemically synthesized GAA needs to be included in broiler chicken feed at a concentration between 0.06% and 0.12%, making it an inflexible raw material with unclarity around optimum dosage and effectiveness. GAA was originally used to reduce the feed conversion ratio (FCR) [23]. Currently, GAA is claimed to spare Arg, allowing the spared Arg to be utilized for protein accretion [11,24]. GAA is also claimed to provide an energy-sparing effect when incorporated at rates of 0.06–0.12% in broiler diets [25,26]. The addition of GAA in broiler diets has shown benefits in increasing Cre levels [20]. GAA addition increases the urinary excretion of GAA, Cre, and creatinine dramatically in a dose-dependent manner (0, 0.6, and 6 g/kg GAA supplementation increases urine content of GAA from 0 to 6.175 and 99.25, Cre from 0 to 1.069 and 28.358 mg/kg, and creatinine from 0 to 1.394 and 63.569 mg/kg of metabolic body weight per day) [20,24].

Via the analysis of performance and carcass yield parameters, this study aimed to determine the Arg requirement of broilers, to determine the Arg-sparing efficacy of GAA, and to determine if there is an energy-sparing effect of GAA in broilers.

## 2. Materials and Methods

### 2.1. Animals and Housing

A cohort of 3000 male Ross 308 broiler chickens were housed in the test facility (Poulpharm BV, Izegem, Belgium). The animals arrived as one-day-old chickens and were placed in 1 m^2^ pens. Throughout the duration of this study, birds were provided ad libitum access to feed and drinking water. Temperature was regulated according to Ross 308 management manual recommendation from 2009 and was reduced from 36 to 22 °C. Care and use of animals were in accordance with the principles and guidelines presented in the Guide for the Care and Use of Agricultural Animals and Research and Teaching [27].

#### 2.1.1. Study Design

Two parallel experiments were conducted to investigate the Arg requirements of broilers and to assess the potential energy and Arg-conserving effects of GAA.

In experiment 1, a total of 1800 birds were randomly allocated to 120 pens, each housing 15 chickens. These pens were subjected to thirteen different dietary treatments (Table 1). Each treatment was replicated nine times (except for the basal diet, which was replicated twelve times), and the feeding trial spanned 35 days. This experiment aimed to elucidate both the Arg requirements and the efficacy of GAA in broilers.

Experiment 2 involved 1200 chickens distributed among five treatments using a completely randomized design. Each treatment was replicated 16 times, with 15 chickens in each replicate. This experiment, conducted over a 35-day trial period, focused on examining the potential energy-saving effect of GAA in broilers. Throughout both experiments, the chickens were provided ad libitum access to feed and water.

#### 2.1.2. Test Diets

L-Arg (Bestamino^TM^ L-Arg, CJ Bio, Seoul, Republic of Korea) is a white, crystalline feed grade AA produced by microbial fermentation, with a minimum content of 98.5% L-Arg. GAA (Creamino^TM^, Alzchem, Trostberg, Germany) is an off-white powder preparation, categorically known as an AA derivative, commercially available at 96% purity. The diets provided during the trial were formulated in pellet form, featuring a size of 2.5 mm for the starter (0–10 days) feed and 3 mm for the grower (10–24 days) and finisher (24–35 days) feed. Notably, these trial diets were devoid of anticoccidials, antimicrobial growth promoters, or enzymes. Feeds were formulated following the Ross 308 recommendations [28].

In experiment 1, incremental levels of 0.06%, 0.12%, 0.18%, 0.30%, 0.45%, and 0.61% of either Arg or GAA were supplemented to a basal diet deficient in Arg (1.2 g Arg/100 g feed, Supplementary Tables S6–S8). Meanwhile, in experiment 2, a comparison was made between a positive control diet (PC) containing standard energy and digestible Arg and two negative control diets (NC)—NC1, wherein the energy was reduced by 50 kcal/kg compared to PC, and NC2, wherein the energy was reduced by 100 kcal/kg compared to PC. Furthermore, supplementation of 0.06% and 0.12% GAA was incorporated into NC1 and NC2, respectively, to compensate for the 50 kcal/kg and 100 kcal/kg energy deficiencies. Detailed information regarding the different treatment groups, Arg, GAA, and energy levels for both experiments is provided in Table 1, Table 2 and Table 3, while the ingredients and nutrient compositions of the diets are outlined in Table 4 and Table 5. Raw materials underwent pre-feed production analysis, and subsequently, formulated feeds were subjected to amino acid content analysis (SGS, Hamburg, Germany). Additionally, Weende analysis and total protein-bound amino acid evaluations (VO EG 152/2009) were conducted. Nutritional compositions of the formulated diets were verified by analyzing the diets, as detailed in Supplementary Tables S1–S3 for experiments 1 and 2.

### 2.2. Evaluation Parameters

The animals’ overall health was under daily surveillance throughout this study. Mortality rates, including birds removed for welfare considerations, were documented, and deceased birds were weighed to ascertain mortality percentages and adjust performance metrics accordingly. At specific intervals, birds and feed within each enclosure were weighed—initially on study day 0 and subsequently at the conclusion of each feeding phase on days 10, 24, and 35. These measurements were used to compute the average standard performance parameters encompassing daily feed intake (DFI), daily weight gain (DWG), and feed conversion ratio (FCR). At the end of the study, on day 35, four birds from each pen were weighed to record their live weight. Then, they were slaughtered and weighed a second time, subsequent to bleeding, evisceration, and removal of feathers, head, neck, and feet. This allowed for the assessment of carcass yield, breast meat, and leg weight.

### 2.3. Statistical Analysis

Statistical analysis was conducted utilizing a linear regression model, comparing all experimental groups against the control diet (Arg 0%). Post hoc pairwise comparisons were undertaken without adjustment methods, and significant differences are denoted in the subsequent tables via compact letter display (*p* < 0.05). The analysis was performed in R (version 3.2.5). Mortality was analyzed using Cox proportional hazard models. Carcass weight, fresh breast meat weight, leg weight, body weight (BW), DWG, ADFI, FCR, as well as calculated slaughter yields and percentages were analyzed with linear regression models. The fit of these models was examined through residual plots. Statistical significance was determined at *p* < 0.05. Data were analyzed in three blocks: T01-T07 (experiment 1): pairwise comparisons with BD as reference + multiple pairwise comparisons. T01 and T08-T13 (experiment 1): pairwise comparisons with T01 as reference + multiple pairwise comparisons. T14-T18 (experiment 2): pairwise comparisons with PC as reference + multiple pairwise comparisons.

Additionally, data from T01-T13 were analyzed using a comparison between quadratic polynomial, linear broken line, quadratic broken line, and exponential asymptotic models. For each model, the Akaike information criterion (AIC) was calculated. The model with the lowest AIC was considered as the optimal model. The selected models were used to determine the optimal requirements of Arg and GAA and the relative bioavailability of Arg versus GAA. The effects of adding GAA to the feed were compared to the effects of adding Arg. Models were fit on data of block 1 (Arg; T01–T07) and block 2 (GAA; T08–T13). The dose and the interaction of the dose and GAA were included as fixed effects. The interaction term indicated if a certain dose of GAA had a different effect compared to the same dose of Arg. Differences between the same dose of GAA and Arg were studied using a linear regression model with dose and the interaction between dose and product as categorical fixed effects. Next, the dose–response of Arg and GAA were modeled with quadratic polynomial models. A direct comparison between Arg and GAA was also made. First, the maximum performance value of GAA and the corresponding GAA dose were identified. Next, the inclusion rate of Arg needed to reach that same performance was determined. Lastly, the relation of these two doses was calculated.

## 3. Results

### 3.1. Experiment 1: Optimizing Response of Broilers with Gradual Increase in Arg and GAA

Based on the performance results (Table 6), digestible Arg levels at 1.14–1.63% in the starter, 0.94–1.49% in the grower, and 0.81–1.36% in the finisher phase significantly increased DWG of the birds compared with the Arg deficient diet in the basal diet group (digestible Arg 1.02, 0.88, and 0.75% from starter to finisher). Moreover, FCR was significantly improved at minimum digestible Arg levels of 1.08, 0.94, and 0.81% at the starter, grower, and finisher phases, respectively.

The minimum digestible Arg levels that provided the highest DWG were 1.20% for the starter, 1.06% for the grower, and 0.87% for the finisher. As for FCR, starter, grower, and finisher diets required 1.47%, 1.18, and 0.87% digestible Arg, respectively, to achieve the best performance (Table 6). Overall, broilers had the highest DWG at 0.30 Arg supplementation and the most efficient feed conversion when Arg was added at 0.45 and 0.61%. These Arg levels translate to dietary Arg content of 1.32–1.63% in the starter phase, 1.18–1.49% in the grower phase, and 1.05–1.36% in the finisher phase (Table 6).

Likewise, supplementation of GAA to the low-Arg diet showed significant improvement in DWG and FCR. In all the measured parameters, the maximum response to GAA supplementation was constantly lower than the maximum response with Arg. The minimum level of GAA inclusion at which a significant increase in DWG was observed in all periods was at 0.06%. On the other hand, FCR was significantly improved at minimum GAA inclusion of 0.18% in the starter and 0.06% in the grower and finisher stages. At 0–35 days, the birds had the greatest DWG improvement at 0.18% GAA supplementation and were most feed efficient when fed diets with 0.12% dietary GAA.

The overall feed intake of broilers significantly increased with increasing levels of digestible Arg. However, a remarkable observation here is the significant depression in ADFI when GAA reached high levels of incorporation into the diets, starting at 0.3% until 0.61% in the starter and at 0.61% in the grower and 0.45% in the finisher phase. Overall, the inclusion of 0.06% GAA significantly increased feed intake, but there was a tendency to decline at 0.45% until a significant reduction was observed at 0.61% GAA. In the same way that ADFI was negatively impacted by high levels of GAA, the DWG was significantly reduced when GAA supplementation reached 0.61%. Interestingly, starter birds did not gain any benefit from adding GAA either or from reducing their feed intake by adding 0.30% GAA or more.

Arginine supplementation improved carcass yield parameters when added at the lowest dosage of 0.06% to the highest dosage of 0.61%, except for leg yield live (LYL) and leg yield carcass (LYC). LYC significantly decreased at 0.12–0.61% inclusion of Arg. At a rate of 0.30% Arg dietary inclusion, the highest yield was attained for carcass yield (CY), slaughter yield (SY), and live slaughter weight (LSW), while breast weight (BW), leg weight (LW), breast yield live (BYL), and breast yield carcass (BYC) were highest at 0.45% Arg inclusion.

GAA supplementation significantly improved carcass characteristics at dietary inclusion rates of 0.06% for LYL and LYC; 0.18% for LSW, CW, LW, and SY; and 0.30% for BW, BYL, and BYC. High levels of GAA at 0.61% resulted in a significant reduction of LSW and CW. For LW, SY, and BW, supplementation with 0.61% GAA had no effect compared to the reference diet without GAA. LYL and LYC yields were significantly reduced at rates of 0.30% and 0.61% and rates above 0.18%, respectively.

The mortality rate was significantly affected by feeding the birds with low-Arg diets. Birds started to improve survivability at 0.06% Arg or 0.30% GAA inclusion (Supplementary Figure S1). The lowest mortality rate was achieved when 0.30% L-Arg or 0.30% and 0.45% GAA were incorporated into the diets.

The data were also analyzed to determine the relative equivalence values between Arg and GAA, as shown in Table 7 and Table 8, Supplementary Tables S4 and S5, and Supplementary Figures S2–S13. The best overall DWG and FCR of broilers were achieved at 0.22% Arg, while 0.29% and 0.33% GAA were required to attain the best DWG and FCR, respectively.

Additionally, a single value representing the relative amount of Arg needed to reach the same performance as the maximum GAA performance that could be identified was calculated. This value can be considered as the relative bioavailability of Arg vs. GAA for that specific parameter. For some parameters, the amount of Arg needed was only 50% or less of the amount of GAA to reach the same performance. Table 9 gives an overview of the relative bioavailability for each parameter at each life phase. To achieve maximum DWG and FCR, the dose equivalences for Arg and GAA were 0.57:1 and 0.78:1, respectively. Thus, Arg is needed at lower amounts compared to GAA to improve the performance of broilers. On average, the equivalence of Arg and GAA in starter, grower, finisher, and overall phases were 0.47:1, 0.84:1, 0.50:1, and 0.53:1, respectively.

### 3.2. Experiment 2: Energy-Sparing Effect of GAA

In the latter segment of the feeding trial, the assessment of the energy-conserving impact of GAA was conducted. The reduction of either 50 or 100 kcal of AMEn across the starter, grower, and finisher diets, alongside GAA supplementation to these energy-depleted diets, exhibited no overall significant influence on BW, DWG, ADFI, or FCR. However, several exceptions were noted. (1) The addition of 0.12% GAA to a −100 kcal AMEn diet resulted in a reduction in average daily feed intake during the starter phase. (2) The −100 kcal diet displayed an elevated FCR compared to the positive control during the starter phase. (3) The inclusion of 0.06% GAA in a −50 kcal AMEn diet led to reduced ADFI, BW, and DWG from D24 to D35. (4) Throughout the entire growth phase, the addition of 0.06% GAA to a −50 kcal AMEn diet resulted in lower ADFI and DWG. (5) Supplementation of 0.06% GAA to a −50 kcal AMEn diet led to reductions in LSW, CW, and LW. Table 10 gives an overview of these results.

## 4. Discussion

Arg-deficient diets impede broiler performance. Such deficiency has been linked to compromised growth and disturbances in blood and muscle levels of energy-related metabolites such as phosphocreatine and creatine in a study by Portocarero and Braun [11]. They further underscored the impact of Arg deficiency on the overall performance of the birds. Notably, birds lack the capacity for sufficient endogenous Arg synthesis due to the absence of carbamoyl phosphate synthetase 1 and exhibit limited renal activity of enzymes necessary for Arg synthesis [29]. Hence, Arg stands as an indispensable amino acid in poultry nutrition, mandating its fulfillment through dietary intake to meet the birds’ essential physiological requirements.

The latest Aviagen guidelines for Ross 308 broilers SID Arg levels are 1.40%, 1.27%, and 1.17% for the starter, grower, and finisher phases, respectively [28]. Conversely, Rostagno [30] proposed varying levels of digestible Arg for different age ranges of broilers, recommending 1.41% for one-week-old broilers, 1.23% for 8–21 days, 1.13% for 22–33 days, 1.05% for 34–42 days, and 0.96% for 34–46 days old. In the initial segment of this investigation, the optimal dietary Arg level was established to exceed the minimum requirements outlined by NRC [31] while closely aligning with the recommendations provided by the breeder company (Aviagen) for each growth phase. Contemporary broilers, owing to advancements in breeding and genetics, exhibit enhanced growth rates, improved feed efficiency, and heightened survivability. However, realizing these objectives necessitates precisely balanced diets to ensure optimal nutrient provision. Recent research indicates that the Arg requirements of birds surpass previous estimations [16], prompting Aviagen to revise and elevate its arginine recommendations in comparison to the Aviagen 2019 guidelines [32].

The progressive inclusion of Arg, starting from the lowest treatments at 1.08%, 0.88%, and 0.75% in starter, grower, and finisher diets, respectively, has demonstrated notable enhancements in body weight gain, feed efficiency, and carcass yield parameters. These findings align with previous studies conducted by Kidd et al. [33], Labadan et al. [34], Corzo et al. [16], Murakami et al. [35], Khajali et al. [36], Xu et al. [37], and Zampiga et al. [38]. Jahanian and Khalifeh-Gholi [39] and Noguiera et al. [40] further corroborated the positive impact of increased dietary Arg on broiler performance.

The concurrent elevation in dietary Arg levels and the observed improvements in broiler performance directly underscore the indispensability of this amino acid in poultry nutrition. Particularly noteworthy is the meta-analysis by Ospina-Rojas et al. [41], synthesizing findings from 94 studies spanning 2001–2019 to ascertain the optimal Arg requirement in broiler diets. This comprehensive analysis revealed a positive correlation between increased Arg intake and enhanced body weight gain, with a significant quadratic regression (*p* = 0.0138) applied to the 94 observations, predicting the daily digestible Arg intake necessary to achieve favorable body weight gain across different growth stages.

The most favorable daily weight gains and feed efficiencies were observed within digestible Arg ranges of 1.32–1.63% for starter, 1.18–1.49% for grower, and 1.05–1.36% for finisher diets. These optimal values surpass the Aviagen recommendations for Ross 308 broilers [28]. Studies by Labadan et al. [34], Corzo et al. [16], Jiao et al. [42], and Noguiera et al. [40] have reported comparable enhancements in breast meat yield attributed to Arg supplementation. Moreover, Noguiera et al. [40] documented a significant increase in carcass yield with a digestible Arg intake of 2.27 g/day in broilers.

The adverse correlation between low-Arg diets and heightened mortality rates in broilers could be elucidated by reduced available energy and essential nutrients, resulting in decreased feed intake. Additionally, Arg plays a pivotal role in bolstering avian immunity; hence, Arg-deficient diets lead to compromised immunity levels, rendering birds more susceptible to health challenges. The gradual addition of Arg ranging from 0.06% to 0.61% to low-Arg diets markedly reduced mortality rates, aligning with findings by Kidd et al. [33] and Corzo et al. [16], who observed a linear decline in broiler mortality rates with graded Arg supplementation. Observations by Tayade et al. [43], Abdukalykova et al. [15], and Jahanian [44] also indicated an amelioration in the overall health status of birds with adequate dietary Arg levels. It is probable that standard corn/wheat–soy diets might not meet the dietary Arg requirements. Therefore, special attention is imperative to ensure optimal levels of digestible Arg in poultry diets. Arg derived from bacterial fermentation processes serves as a viable means to achieve a well-balanced Arg concentration in chicken diets without a substantial increase in crude protein levels.

There exists ongoing research aimed at understanding the Arg-sparing potential of GAA. In this study, supplementation of GAA to low-Arg diets optimized DWG and FCR at inclusion rates of 0.18% and 0.12%, respectively. However, the performance outcomes with GAA consistently trailed behind those achieved with Arg supplementation. Dilger et al. [45] demonstrated that 0.12% GAA (not 0.06% GAA) elicited better weight gain responses than a basal diet; however, the performance results fell significantly below those of their Arg-supplemented group. Furthermore, neither GAA nor Cre exhibited a complementary effect when added on top of an Arg-supplemented group.

Dilger et al. [45] conducted a dose–response trial using two basal diets—one deficient in Arg and the other deficient in Arg but supplemented with 0.12% GAA. Weight gain and gain-to-feed ratio showed improvement under severely deficient Arg conditions. However, close to established Arg recommendations, adding 0.12% GAA did not create further differences. De Groot [5] demonstrated that while GAA improved the gain-to-feed ratio, Arg improved not only gain-to-feed but also DWG and BW.

Portocarero and Braun [11] attempted to define Arg-to-GAA equivalence values from various broiler studies, although none were bioefficacy studies. On average, an improvement in DWG was reported at ratios ranging from 1:1 to as high as 3:1, while for FCR, a range of 0.77:1 to 3:1 was reported. This study presents, for the first time, a bioefficacy of 56% based on a dose–response study using graded levels of Arg and GAA.

In this study, improvement in DWG and FCR was observed at 0.18% and 0.12% GAA, respectively. Calculated equivalency values between Arg and GAA suggest that only 57% and 78% of Arg is required to produce similar impacts on DWG and FCR relative to GAA. These values are lower than estimates provided by Ringel et al. [46] and Scharch et al. [47], who reported improvements in DWG at an Arg-to-GAA ratio of 1.16:1 and enhanced FCR at a ratio of 0.77:1.

The observed ratio of Arg-to-GAA in this study to improve DWG was found at 0.57:1, differing from previous findings by Dilger et al. [45], who reported replacement ratios between 1:1 and 2:1. In a similar study by De Groot et al. [5] involving Arg-deficient diets supplemented with or without GAA, they suggested replacement factor for BWG was 1.3:1 (Arg:GAA) and over 1.3:1 for FCR response, noting that these ratios were calculated not derived from efficacy studies.

Moreover, studies by Dilger et al. [45] highlighted that at low dietary Arg levels, Arg supplementation could not be equally replaced by GAA. Portocarero and Braun [11] supported this, indicating that severe Arg deficiency limits GAA’s ability to fully compensate for the deficit, whereas if Arg is adequate, GAA’s effects are more pronounced. However, these conclusions are not based on bioefficacy studies. There is a lack of comparison between different levels of Arg and GAA under less deficient Arg conditions.

The research into the energy-sparing effect of GAA is notably limited compared to the extensive research on its Arg-sparing effect. Within this study, a reduction of either 50 or 100 kcal of metabolizable energy in broiler diets did not significantly affect the overall performance of the birds. These findings align with reports by Abudabos et al. [48] and Heger et al. [26], indicating that DWG and FCR remained largely unaffected by dietary energy levels. Broilers’ response to varying energy levels appears contingent upon the degree of increase or reduction. For instance, as exemplified by Dozier and Gehring [49], birds showed no response to a reduction of 25–50 kcal/kg in AMEn.

Energy evaluation systems exhibit considerable variability, and feed formulation accuracy concerning energy remains relatively low. The actual metabolizable energy of raw materials, influenced by their physical and chemical properties, contrasts with the calculated AMEn, which often does not reflect the true nature of final feed properties. Consequently, the marginal to negligible effects observed with the reduction of 50 to 100 kcal in energy on broilers could be attributed to these discrepancies.

While some studies by Abudabos et al. [48], Heger et al. [26], Ale Saheb Fosoul et al. [50], and Ceylan et al. [51] described positive growth and/or feed efficiency responses with GAA supplementation in reduced-energy diets, this study did not clearly demonstrate such effects, aside from a few exceptional reductions in performance parameters upon GAA addition to an energy-deficient diet. Further investigations are warranted to distinctly determine whether GAA exhibits a negative or positive impact on growth performance parameters under varying energy levels.

Regarding the findings from the two conducted experiments, a noticeable decrease in feed intake was observed when Arg was deficient or when GAA was supplemented at levels exceeding the recommended dosage of 0.06–0.12%. In experiment 1, where the Arg-sparing effect of GAA was assessed, low-Arg diets led to diminished feed intake among the birds. This parallels the observations of Melare et al. [52], who indicated that reduced feed intake might stem from the dilution of feed due to the increased utilization of bulky ingredients in formulating low-Arg diets. Additionally, birds might strive to maintain their thermal balance as the ratio of energy to amino acids becomes disrupted in such diets.

Another study by Nogueira et al. [40] corroborated this notion, further explicating that the depression in feed consumption reflects the birds’ efforts to regulate their thermal balance when the balance between energy and amino acids in feeds is altered. Birds tend to decrease their feed intake to mitigate imbalances, given that the surplus energy consumed cannot be channeled into other metabolic pathways. This trend is particularly noticeable in modern broiler strains selected for lean tissue deposition, resulting in a reduced capacity for body lipid storage [40].

The impact of GAA on the feed intake of birds fed low-Arg diets revealed a pattern where broilers began consuming less feed when GAA was supplemented at levels exceeding 0.3% in an Arg-deficient diet. Notably, a significant effect on growth rate was observed at 0.61% GAA inclusion in low-Arg diets, aligning with findings by Dilger et al. [45], who documented a sudden decline in feed intake when birds were fed Arg-deficient diets supplemented with GAA within the range of 0.39–0.78%.

Similarly, in experiment 2, exploring the energy-sparing effect of GAA, when GAA was supplemented at 0.06% or 0.12% to energy-reduced diets, a parallel reduction in feed intake was observed. This echoes results from Abudabos et al. [48], indicating that irrespective of dietary energy levels, GAA supplementation led to decreased feed intake. Heger et al. [26] reported a notable reduction in feed intake when GAA was added to the diet at 0.06%. A similar observation was also made by Mousavi et al. [25]. However, Michiels et al. [23] reported a slight increase in feed intake in broilers fed diets supplemented with 0.06% or 0.12% GAA, contrasting with Ringel et al. [46], who observed no effect of GAA on feed intake. It is worth noting that the reduction in feed intake associated with GAA supplementation might partly arise due to the potentially toxic effects of high GAA levels in feed, as mentioned by EFSA [19,53].

The need for further studies on the negative impact of GAA, particularly on younger birds (1–10 days), becomes apparent as observed benefits from GAA supplementation were absent in these birds. Additionally, the negative influence of GAA on voluntary feed intake, as suggested by Heger et al. [26], should be investigated further.

Considering the declining trend in feed intake associated with GAA supplementation, it becomes evident that GAA can significantly affect the nutrient intake of birds. Careful consideration is advised, especially when diets are already deficient in energy or amino acids. In feeding trials aiming to demonstrate the Arg or energy-sparing effects of GAA, caution is warranted as compromised performance may arise if birds exhibit an aversion to the feed.

## 5. Conclusions

Based on the outcomes derived from this study, optimal performance in broilers was achieved when birds were provided with higher SID Arg levels compared to the recommended requirements outlined by Aviagen in 2019 or 2022 [28,32]. Arg-deficient diets resulted in subpar performance among the birds. While supplementation of Arg improved performance, the Arg-sparing effect of GAA in promoting the growth rate and feed efficiency of broilers was calculated at an equivalency ratio of Arg:GAA of approximately 0.57:1 and 0.78:1, respectively.

It is noteworthy that birds supplemented with GAA could not achieve the maximum performance levels attained through the addition of supplementary Arg across the investigated parameters. On average, the efficacy of GAA in sparing Arg was demonstrated at approximately 59.6%. Moreover, neither the reduction in energy levels of the diets at 50 and 100 kcal/kg nor the supplementation of GAA significantly influenced the overall performance of the birds in the study.

## Figures and Tables

**Table 1:** Overview of different treatment groups in experiment 1 (T01–T13) and experiment 2 (T14–T18).

Treatment Group	Diet	Replicates	Birds/Replicate	Starter Dose%	Starter Total%	Grower Dose%	Grower Total%	Finisher Dose%	Finisher Total%
T01	A basal diet deficient in Arg (BD)	12	15	0	1.02	0	0.88	0	0.75
T02	0.06% SID Arg (below ROSS 308)	9	15	0.06	1.08	0.06	0.94	0.06	0.81
T03	0.12% SID Arg (below ROSS 308)	9	15	0.12	1.14	0.12	1	0.12	0.87
T04	0.18% SID Arg (below ROSS 308)	9	15	0.18	1.20	0.18	1.06	0.18	0.93
T05	0.30% SID Arg (around ROSS 308)	9	15	0.30	1.32	0.30	1.18	0.30	1.05
T06	0.45% SID Arg (above ROSS 308)	9	15	0.45	1.47	0.45	1.25	0.45	1.20
T07	0.61% SID Arg (above ROSS 308)	9	15	0.61	1.63	0.61	1.49	0.61	1.36
T08	0.06% GAA	9	15	0.06		0.06		0.06	
T09	0.12% GAA	9	15	0.12		0.12		0.12	
T10	0.18% GAA	9	15	0.18		0.18		0.18	
T11	0.30% GAA	9	15	0.30		0.30		0.30	
T12	0.45% GAA	9	15	0.45		0.45		0.45	
T13	0.61% GAA	9	15	0.61		0.61		0.61	
T14	Control diet (normal energy and AAs)	16	15	2960 kcal	1.37	3050 kcal	1.23	3150 kcal	1.10
T15	T14 minus 50 kcal energy	16	15	2910 kcal	1.37	3000 kcal	1.23	3100 kcal	1.10
T16	T14 minus 100 kcal energy	16	15	2860 kcal	1.37	2950 kcal	1.23	3050 kcal	1.10
T17	T15 + 600 g GAA	16	15						
T18	T16 + 1200 g GAA	16	15						

GAA: guanidinoacetic acid. Arg: arginine. Starter: 0–10 days. Grower: 10–24 days. Finisher: 24–35 days. T1–T13 are used in experiment 1, and T14–T18 are used in experiment 2.

**Table 2:** Standardized ileal digestible Arg levels of dietary treatments (experiment 1).

**Diet**	**L-Arg Inclusion Rate, %**						
	**0**	**0.06**	**0.12**	**0.18**	**0.30**	**0.45**	**0.61**
Starter	1.02	1.08	1.14	1.20	1.32	1.47	1.63
Grower	0.88	0.94	1.00	1.06	1.18	1.25	1.49
Finisher	0.75	0.81	0.87	0.93	1.05	1.20	1.36
	**GAA Inclusion Rate, %**						
		**0.06**	**0.12**	**0.18**	**0.30**	**0.45**	**0.61**
Starter		1.02	1.02	1.02	1.02	1.02	1.02
Grower		0.88	0.88	0.88	0.88	0.88	0.88
Finisher		0.75	0.75	0.75	0.75	0.75	0.75

GAA: guanidinoacetic acid. Arg: arginine. Starter: 0–10 days. Grower: 10–24 days. Finisher: 24–35 days.

**Table 3:** Energy, crude protein, and arginine levels of dietary treatments (experiment 2).

Diet	AMEn, kcal/kg			% CP	% Arg
	Control	−50 AMEn	−100 AMEn		
Starter	2960	2910	2860	22.80	1.37
Grower	3050	3000	2950	21.18	1.23
Finisher	3150	3100	3050	18.60	1.10

AMEn: apparent metabolizable energy. Starter: 0–10 days. Grower: 10–24 days. Finisher: 24–35 days.

**Table 4:** Ingredients and nutrient composition of basal broiler diets (experiment 1).

Ingredient Name	Starter (0–10 Days)	Grower (10–24 Days)	Finisher (24–35 Days)
Corn	50.00	55.00	69.87
Soybean Meal	20.36	14.38	8.86
Rape Seed Meal	8.00	10.00	5.18
Corn Starch	6.00		
Corn Gluten Meal	5.55	5.10	10.00
Corn Gluten Feed	2.78	6.76	
Soy Oil	1.98	4.27	1.34
DCP	1.60	0.95	1.14
Limestone	1.06	0.92	0.96
L-Lysine HCL (99%, CJ BIO)	0.61	0.59	0.62
Broiler Premix	0.50	0.50	0.50
Salt	0.37	0.37	0.39
L-Methionine (99%, CJ BIO)	0.37	0.32	0.24
L-Threonine (80%, CJ BIO)	0.29	0.26	0.21
L-Glycine (99%, CJ BIO)	0.27	0.33	0.51
L-Isoleucine (90%, CJ BIO)	0.15	0.16	0.11
L-Valine (96.5%, CJ BIO)	0.11	0.08	0.06
L-Tryptophan (98%, CJ BIO)	0.02	0.02	0.04
**Calculated Nutrient Composition %**			
AMEn Broiler (kcal/kg)	2960	3050	3150
Crude Protein	21.43	20.12	18.11
Crude Fat	5.49	8.12	5.56
Crude Fiber	3.32	3.70	2.89
Ash	6.15	5.41	4.95
Calcium	0.90	0.70	0.70
Available Phosphorous	0.42	0.32	0.32
SID Lysine	1.28	1.15	1.03
SID Methionine	0.65	0.59	0.52
SID Met+Cys	0.95	0.87	0.80
SID Arginine	1.02	0.88	0.75
SID Threonine	0.86	0.77	0.69
SID Leucine	1.63	1.48	1.70
SID Isoleucine	0.86	0.78	0.71
SID Valine	0.96	0.87	0.78
SID Tryptophan	0.20	0.18	0.16
SID Phenylalanine	0.86	0.78	0.75
SID Histidine	0.47	0.44	0.39
Choline	1439	1492	1030
Starch	34.63	38.07	46.94

DCP: dicalcium phosphate; SID: standardized ileal digestible; Met: methionine; Cys: cysteine; AMEn: apparent metabolizable energy corrected for nitrogen. Empty cells are equal to zero.

**Table 5:** Ingredients and nutrient composition of basal broiler diets (experiment 2).

Ingredient Name	Starter (0–10 Days)			Grower (10–24 Days)			Finisher (24–35 Days)		
	PC	NC1	NC2	PC	NC1	NC2	PC	NC1	NC2
Corn	39.70	41.00	42.30	38.40	39.70	40.99	40.21	41.51	42.81
Soybean Meal	33.24	33.00	32.77	29.16	28.93	28.69	21.70	21.46	21.23
Wheat	20.00	20.00	20.00	25.00	25.00	25.00	30.00	30.00	30.00
Soy oil	2.32	1.25	0.18	3.36	2.29	1.22	3.99	2.91	1.84
DCP	1.63	1.63	1.62	1.08	1.08	1.07	1.15	1.14	1.14
Limestone	1.08	1.08	1.08	0.91	0.92	0.92	0.92	0.93	0.93
Broiler Oremix	0.50	0.50	0.50	0.50	0.50	0.50	0.50	0.50	0.50
Salt	0.38	0.38	0.38	0.39	0.39	0.39	0.39	0.40	0.40
L-Met (99%, CJ BIO)	0.37	0.37	0.37	0.32	0.32	0.31	0.30	0.30	0.30
L-Lys HCL (99%, CJ BIO)	0.37	0.37	0.38	0.32	0.32	0.33	0.38	0.39	0.39
L-Thr (80%, CJ BIO)	0.23	0.23	0.23	0.18	0.18	0.18	0.20	0.20	0.20
L-Arg (98.5%, CJ BIO)	0.12	0.13	0.13	0.09	0.09	0.09	0.16	0.16	0.16
L-Val (96.5%, CJ BIO)	0.04	0.04	0.04	0.01	0.01	0.01	0.04	0.04	0.04
L-Ile (90%, CJ BIO)	0.03	0.03	0.03	0.01	0.01	0.01	0.06	0.06	0.07
L-Gly (99%, CJ BIO)	0.00	0.00	0.00	0.28	0.28	0.28	0.00	0.00	0.00
**Nutrient composition %**									
AMEn Broiler (kcal/kg)	2960	2910	2860	3050	3000	2950	3150	3100	3050
Crude Protein	22.80	22.80	22.80	21.18	21.18	21.18	18.59	18.59	18.59
Crude Fat	5.27	4.25	3.24	6.29	5.28	4.26	6.97	5.96	4.94
Crude Fiber	2.67	2.69	2.71	2.62	2.64	2.67	2.53	2.55	2.57
Ash	6.40	6.40	6.39	5.53	5.53	5.52	5.25	5.25	5.24
Calcium	0.90	0.90	0.90	0.70	0.70	0.70	0.70	0.70	0.70
Available Phosphorous	0.42	0.42	0.42	0.32	0.32	0.32	0.32	0.32	0.32
SID Lysine	1.28	1.28	1.28	1.15	1.15	1.15	1.03	1.03	1.03
SID Methionine	0.64	0.63	0.63	0.57	0.57	0.57	0.53	0.53	0.52
SID Met+Cys	0.95	0.95	0.95	0.87	0.87	0.87	0.80	0.80	0.80
SID Arginine	1.37	1.37	1.37	1.23	1.23	1.23	1.10	1.10	1.10
SID Threonine	0.86	0.86	0.86	0.77	0.77	0.77	0.69	0.69	0.69
SID Leucine	1.54	1.54	1.54	1.43	1.44	1.44	1.25	1.25	1.25
SID Isoleucine	0.86	0.86	0.86	0.78	0.78	0.78	0.71	0.71	0.71
SID Valine	0.96	0.96	0.96	0.87	0.87	0.87	0.78	0.78	0.78
SID Tryptophan	0.23	0.23	0.23	0.21	0.21	0.21	0.18	0.18	0.17
SID Phenylalanine	0.93	0.93	0.93	0.86	0.86	0.86	0.73	0.73	0.73
SID Histidine	0.50	0.50	0.50	0.47	0.47	0.47	0.40	0.40	0.40
Choline	1313	1314	1315	1233	1234	1235	1077	1079	1080
Starch	39.78	40.60	41.42	41.84	42.65	43.47	45.70	46.52	47.34

DCP: dicalcium phosphate; SID: standardized ileal digestible; Met: methionine; Cys: cysteine; AMEn: apparent metabolizable energy corrected for nitrogen. Empty cells are equal to zero. PC: positive control; NC: negative control.

**Table 6:** Summary of performance and carcass yield of broilers fed low-arginine diets supplemented with L-Arg or GAA at different rates from 0 to 35 days of age (*p* < 0.05) (experiment 1).

**Parameter**	**% Supplemental Arginine**							
	**0**	**0.06**	**0.12**	**0.18**	**0.30**	**0.45**	**0.61**	
Day 0–10								SE
Initial wt., g	41.6 ^b^	41.8 ^ab^	41.5 ^b^	41.6 ^ab^	41.8 ^ab^	42.2 ^a^	41.7 ^ab^	0.2
Final wt., g	265.0 ^d^	273.1 ^cd^	282.6 ^bc^	287.0 ^ab^	287.0 ^ab^	296.0 ^da^	291.3 ^ab^	28
DWG, g	22.3 ^c^	23.0 ^c^	24.1 ^b^	24.5 ^ab^	24.5 ^ab^	25.3 ^a^	25.0 ^ab^	0.4
ADFI, g	25.9	25.6	26.3	26.6	26.0	26.2	25.9	0.4
FCR	1.16 ^a^	1.11 ^b^	1.09 ^bc^	1.08 ^cd^	1.06 ^de^	1.04 ^e^	1.04 ^e^	0.01
Day 10–24								
BW, g	1042 ^d^	1149 ^c^	1225 ^b^	1273 ^ab^	1305 ^a^	1320 ^a^	1293 ^a^	3.7
DWG, g	54.3 ^d^	62.6 ^c^	67.0 ^b^	70.4 ^a^	72.3 ^a^	72.6 ^a^	71.6 ^a^	1.1
ADFI, g	88.3 ^b^	89.0 ^b^	93.1 ^ab^	94.4 ^a^	95.5 ^a^	94.4 ^a^	92.3 ^ab^	1.8
FCR	1.63 ^a^	1.43 ^b^	1.39 ^bc^	1.34 ^cd^	1.32 ^d^	1.30 ^d^	1.29 ^d^	0.02
Day 24–35								
BW, g	1644 ^d^	1949 ^c^	2143 ^b^	2187 ^ab^	2237 ^ab^	2236 ^a^	2184 ^a^	17
DWG, g	51.5 ^c^	71.6 ^b^	82.9 ^a^	83.0 ^a^	84.8 ^a^	83.2 ^a^	80.0 ^a^	2.1
ADFI, g	136.2 ^d^	158.4 ^c^	171.9 ^a^	170.4 ^ab^	170.9 ^ab^	169.1 ^ab^	164.4 ^bc^	2.5
FCR	2.72 ^a^	2.22 ^b^	2.08 ^b^	2.05 ^b^	2.02 ^b^	2.04 ^b^	2.06 ^b^	0.07
Day 0–35								
DWG, g	44.2 ^d^	54.1 ^c^	59.6 ^b^	61.1 ^ab^	62.5 ^a^	62.2 ^a^	60.7 ^ab^	0.9
ADFI, g	84.8 ^c^	92.6 ^b^	98.4 ^a^	98.6 ^a^	99.2 ^a^	97.8 ^a^	95.5 ^ab^	1.4
FCR	1.93 ^a^	1.71 ^b^	1.65 ^c^	1.61 ^cd^	1.59 ^d^	1.57 ^d^	1.57 ^d^	0.02
Mortality, %	7.2 ^a^	1.5 ^b^	1.5 ^b^	2.2 ^ab^	0.7 ^b^	3.7 ^ab^	3.3 ^ab^	0.11
Carcass Yield								
LSW, g	1652 ^d^	1970 ^c^	2138 ^b^	2167 ^b^	2235 ^a^	2241 ^a^	2199 ^ab^	22
CW, g	1210 ^e^	1468 ^d^	1593 ^c^	1641 ^bc^	1704 ^a^	1690 ^ab^	1675 ^ab^	19
BW, g	264 ^e^	332 ^d^	385 ^c^	412 ^b^	436 ^a^	438 ^a^	423 ^ab^	8
LW, g	476 ^d^	568 ^c^	613 ^b^	616 ^ab^	628 ^ab^	637 ^a^	632 ^ab^	8
SY, %	73.1 ^c^	74.5 ^b^	74.5 ^b^	75.8 ^a^	76.3 ^a^	75.5 ^ab^	76.2 ^a^	0.5
BYL, %	15.9 ^d^	16.8 ^c^	18.0 ^b^	19.0 ^a^	19.5 ^a^	19.6 ^a^	19.2 ^a^	0.3
BYC, %	21.8 ^c^	22.7 ^c^	24.2 ^b^	25.0 ^ab^	25.5 ^a^	25.9 ^a^	25.3 ^ab^	0.4
LYL, %	28.8 ^a^	28.8 ^ab^	28.7 ^bc^	28.5 ^de^	28.1 ^e^	28.5 ^cd^	28.8 ^cd^	0.3
LYC, %	39.4 ^a^	38.7 ^ab^	38.5 ^bc^	37.6 ^de^	36.8 ^e^	37.8 ^cd^	37.8 ^cd^	0.3
**Parameter**	**% GAA**							
	**0**	**0.06**	**0.12**	**0.18**	**0.30**	**0.45**	**0.61**	
Day 0–10								SE
Initial wt., g	41.6 ^bc^	42.3 ^a^	41.4 ^c^	42.1 ^ab^	41.5 ^bc^	41.7 ^ac^	41.4 ^c^	0.2
Final wt., g	265.0 ^c^	278.9 ^ab^	273.3 ^ac^	281.5 ^a^	268.8 ^bc^	252.6 ^d^	237.8 ^e^	28
DWG, g	22.3 ^c^	23.7 ^ab^	23.2 ^ac^	23.9 ^a^	22.6 ^bc^	21.1 ^d^	19.6 ^e^	0.4
ADFI, g	25.9 ^a^	26.4 ^a^	26.2 ^a^	26.3 ^a^	24.3 ^b^	23.1 ^bc^	22.1 ^c^	0.5
FCR	1.16 ^a^	1.12 ^ac^	1.13 ^ab^	1.10 ^bc^	1.08 ^c^	1.10 ^bc^	1.13 ^ac^	0.02
Day 10–24								
BW, g	1042 ^d^	1189 ^bc^	1225 ^ab^	1256 ^a^	1235 ^ab^	1164 ^c^	913 ^e^	3.9
DWG, g	54.3 ^d^	64.1 ^c^	67.9 ^ab^	69.3 ^a^	69.0 ^a^	64.8 ^bc^	47.4 ^e^	1.1
ADFI, g	88.3 ^a^	91.5 ^a^	91.3 ^a^	92.4 ^a^	91.2 ^a^	87.0 ^a^	71.7 ^b^	2.0
FCR	1.63 ^a^	1.43 ^c^	1.35 ^d^	1.33 ^d^	1.32 ^d^	1.34 ^d^	1.52 ^b^	0.03
Day 24–35								
BW, g	1644 ^d^	1934 ^c^	2000 ^bc^	2092 ^a^	2055 ^ab^	1937 ^c^	1568 ^e^	17
DWG, g	51.5 ^d^	66.5 ^b^	70.3 ^ab^	75.9 ^a^	74.5 ^a^	70.2 ^ab^	59.6 ^c^	2.0
ADFI, g	136.2 ^d^	154.0 ^bc^	154.7 ^ac^	161.9 ^a^	157.6 ^ab^	149.2 ^c^	134.9 ^d^	2.6
FCR	2.72 ^a^	2.33 ^b^	2.20 ^bc^	2.13 ^bc^	2.12 ^c^	2.13 ^bc^	2.27 ^bc^	0.07
Day 0–35								
DWG, g	44.2 ^d^	53.0 ^c^	55.7 ^ab^	58.2 ^a^	57.4 ^a^	53.9 ^bc^	43.0 ^d^	0.9
ADFI, g	84.8 ^c^	91.6 ^ab^	92.2 ^ab^	94.9 ^a^	92.8 ^a^	88.0 ^bc^	76.6 ^d^	1.5
FCR	1.93 ^a^	1.73 ^b^	1.65 ^c^	1.63 ^c^	1.62 ^c^	1.63 ^c^	1.78 ^b^	0.02
Mortality, %	7.2 ^a^	5.9 ^ab^	2.2 ^ab^	2.2 ^ab^	1.5 ^b^	1.5 ^b^	4.4 ^ab^	0.12
Carcass Yield								
LSW, g	1652 ^d^	1926 ^c^	2017 ^ab^	2070 ^a^	2063 ^a^	1966 ^bc^	1616 ^d^	22
CW, g	1210 ^d^	1418 ^c^	1489 ^ab^	1542 ^a^	1534 ^a^	1452 ^bc^	1151 ^e^	20
BW, g	264 ^d^	314 ^c^	350 ^b^	377 ^a^	382 ^a^	353 ^b^	275 ^d^	8
LW, g	476 ^c^	566 ^ab^	579 ^a^	586 ^a^	576 ^ab^	555 ^b^	444 ^d^	8

GAA: guanidinoacetic acid; Arg: arginine; DWG: daily weight gain; ADFI: average daily feed intake; FCR: feed conversion ratio; BW: body weight or breast weight (within the context of carcass yield); LSW: live slaughter weight; CW: carcass weight; LW: leg yield. Significant differences are indicated via compact letter display in superscript (*p* < 0.05).

**Table 7:** Overview of the supplemented Arg or GAA level at which the best performance was reached in the model showing the best fit (experiment 1).

**Parameter**	**L-Arginine Supplementation at Which the Maximum Performance Was Attained**			
	**Starter** **(D0–10)**	**Grower** **(D10–24)**	**Finisher** **(D24–35)**	**Overall** **(D0–35)**
Body weight	0.45%	0.19%	0.13%	-
Daily weight gain	0.33%	0.31%	0.09%	0.22%
Daily feed intake	0.14%	0.31%	0.09%	0.10%
Feed conversion ratio (lowest FCR)	0.43%	0.24%	0.08%	0.22%
Live slaughter weight	-	-	-	0.23%
Carcass weight	-	-	-	0.26%
Breast weight	-	-	-	0.37%
Leg weight	-	-	-	0.21%
Slaughter yield	-	-	-	0.20%
Breast yield live	-	-	-	0.21%
Breast yield carcass	-	-	-	0.21%
Leg yield live	-	-	-	0%
Leg yield carcass	-	-	-	0%
**Parameter**	**GAA Supplementation at Which the Maximum Performance Was Attained**			
	**Starter** **(D0–10)**	**Grower** **(D10–24)**	**Finisher** **(D24–35)**	**Overall** **(D0–35)**
Body weight	0.16%	0.26%	0.28%	-
Daily weight gain	0.16%	0.27%	0.32%	0.29%
Daily feed intake	0%	0.2%	0.28%	0.25%
Feed conversion ratio (lowest FCR)	0.34%	0.32%	0.08%	0.33%
Live slaughter weight	-	-	-	0.29%
Carcass weight	-	-	-	0.28%
Breast weight	-	-	-	0.30%
Leg weight	-	-	-	0.27%
Slaughter yield	-	-	-	0.26%
Breast yield live	-	-	-	0.35%
Breast yield carcass	-	-	-	0.38%
Leg yield live	-	-	-	0%
Leg yield carcass	-	-	-	0%

GAA: guanidinoacetic acid; Arg: arginine.

**Table 8:** Overview of the highest dosage rate of Arg at which no difference in performance with GAA was observed (experiment 1).

Parameter	Arginine Concentration at Which the Maximum Is Reached			
	Starter (D0–10)	Grower (D10–24)	Finisher (D24–35)	Overall (D0–35)
Body weight	0.18%	0.18%	0.06%	-
Daily weight gain	0.18%	0.18%	BD	0.06%
Daily feed intake	0.18%	0.30%	0.06%	0.06%
Feed conversion ratio (lowest FCR)	0.06%	0.45%	0.45%	0.30%
Live slaughter weight	-	-	-	0.06%
Carcass weight	-	-	-	0.06%
Breast weight	-	-	-	0.06%
Leg weight	-	-	-	0.06%
Slaughter yield	-	-	-	0.18%
Breast yield live	-	-	-	0.18%
Breast yield carcass	-	-	-	0.30%
Leg yield live	-	-	-	0.45%
Leg yield carcass	-	-	-	0.45%

GAA: guanidinoacetic acid; Arg: arginine.

**Table 9:** Overview of the percentage Arg needed, relative to the dose of GAA that resulted in maximum performance with GAA supplementation (experiment 1).

Parameter	% Arg vs. GAA			
	Starter (D0–10)	Grower (D10–24)	Finisher (D24–35)	Overall (D0–35)
Body weight	46%	77%	55%	-
Daily weight gain	46%	84%	44%	57%
Daily feed intake	Inf.	73%	39%	44%
Feed conversion ratio (lowest FCR)	50%	102%	61%	78%
Live slaughter weight	-	-	-	56%
Carcass weight	-	-	-	53%
Breast weight	-	-	-	51%
Leg weight	-	-	-	56%
Slaughter yield	-	-	-	36%
Breast yield live	-	-	-	48%
Breast yield carcass	-	-	-	51%

GAA: guanidinoacetic acid; Arg: arginine.

**Table 10:** Summary of performance and carcass yield of broilers fed energy-deficient diets supplemented with GAA at 0.06 or 0.12% (*p* < 0.05) (experiment 2).

Parameter	Control	−50 kcal/kg	−100 kcal/kg	−50 kcal/kg +0.06% GAA	−100 kcal/kg +0.12% GAA	
Day 0–10						SE
Initial wt., g	41.7	41.7	41.7	41.8	41.7	0.2
Final wt., g	302.2	303.3	302.1	304.2	301.2	29
DWG, g	25.9	26.1	25.8	26	25.6	0.3
ADFI, g	27.7 ^ab^	28.2 ^ab^	28.7 ^a^	27.8 ^ab^	27.7 ^b^	0.3
FCR	1.07 ^b^	1.08 ^b^	1.11 ^a^	1.07 ^b^	1.08 ^b^	0.01
Day 10–24						
BW, g	1355	1374	1358	1359	1358	2.9
DWG, g	74.8	76.0	75.4	75.2	75.5	0.7
ADFI, g	98.5	100.5	100.4	98.9	99.7	0.9
FCR	1.32	1.32	1.33	1.32	1.32	0.01
Day 24–35						
BW, g	2396 ^ab^	2460 ^a^	2412 ^ab^	2369 ^b^	2384 ^ab^	10
DWG, g	94.5 ^ab^	98.4 ^a^	95.8 ^ab^	91.4 ^b^	93.0 ^ab^	2.2
ADFI, g	164.4 ^ab^	168.8 ^a^	167.8 ^a^	159.7 ^b^	164.4 ^ab^	2.4
FCR	1.75	1.72	1.76	1.77	1.77	0.03
Day 0–35						
DWG, g	66.8 ^ab^	68.4 ^a^	67.4 ^ab^	65.9 ^b^	66.5 ^ab^	0.8
ADFI, g	98.6 ^ab^	100.7 ^a^	100.7 ^a^	97.0 ^b^	99.0 ^ab^	1.0
FCR	1.48	1.47	1.49	1.48	1.49	0.01
Mortality, %	2.1	4.6	3.3	4.6	4.2	0.14
Carcass Yield						
LSW, g	2438 ^ab^	2476 ^a^	2439 ^ab^	2366 ^c^	2408 ^bc^	18
CW, g	1848 ^a^	1882 ^a^	1848 ^a^	1800 ^b^	1842 ^ab^	16
BW, g	521	523	527	512	533	8
LW, g	671 ^bc^	692 ^a^	677 ^ab^	654 ^c^	671 ^bc^	7

GAA: guanidinoacetic acid; Arg: arginine. DWG: daily weight gain; ADFI: average daily feed intake; FCR: feed conversion ratio; BW: body weight or breast weight (within the context of carcass yield); LSW: live slaughter weight; CW: carcass weight; LW: leg yield. Significant differences are indicated via compact letter display in superscript (*p* < 0.05).

## Data Availability

The original contributions presented in this study are included in this article/Supplementary Material. Further inquiries can be directed to the corresponding author.

## Supplementary Materials

The following supporting information can be downloaded at https://www.mdpi.com/article/10.3390/ani15010004/s1. Figure S1: Kaplan Meier survival graphs for the Arg (top left) and GAA (top right) supplementation groups (experiment 1), and the energy deficient diets + GAA (bottom) supplementation groups (experiment 2); Figure S2: Overview of the different fitted models for feed conversion ratio for Arg supplementation groups (experiment 1), during starter (top left), grower (top right) and finisher (bottom left) phase and overall (bottom right); Figure S3: Overview of the different fitted models for daily weight gain for Arg supplementation groups (experiment 1), during starter (top left), grower (top right) and finisher (bottom left) phase and overall (bottom right); Figure S4: Overview of the different fitted models for body weight for Arg supplementation groups (experiment 1), during starter (top left), grower (top right) and finisher (bottom left) phase; Figure S5: Overview of the different fitted models for daily feed intake for Arg supplementation groups Supplementary Figure S4. Overview of the different fitted models for body weight for Arg supplementation groups (experiment 1), during starter (top left), grower (top right) and finisher (bottom left) phase and overall (bottom right); Figure S6: Overview of the different fitted models for absolute slaughter parameters for Arg supplementation groups (experiment 1); Figure S7: Overview of the different fitted models for relative slaughter parameters for Arg supplementation groups (experiment 1); Figure S8: Overview of the different fitted models for feed conversion for GAA supplementation groups (experiment 1), during starter (top left), grower (top right) and finisher (bottom left) phase and overall (bottom right); Figure S9: Overview of the different fitted models for daily weight gain for GAA supplementation groups (experiment 1), during starter (top left), grower (top right) and finisher (bottom left) phase and overall (bottom right); Figure S10: Overview of the different fitted models for body weight for GAA supplementation groups (experiment 1), during starter (top left), grower (top right) and finisher (bottom left) phase; Figure S11: Overview of the different fitted models for daily feed intake for GAA supplementation groups (experiment 1), during starter (top left), grower (top right) and finisher (bottom left) phase and overall (bottom right); Figure S12: Overview of the different fitted models for absolute slaughter parameters for GAA supplementation groups (experiment 1); Figure S13: Overview of the different fitted models for relative slaughter parameters for GAA supplementation groups (experiment 1); Table S1: Proximate and Gross Energy components of broiler starter; Table S2: Proximate and Gross Energy components of broiler grower; Table S3: Proximate and Gross Energy components of broiler finisher; Table S4: Overview of the predicted arginine doses needed to attain maximal, 99% and 95% of maximal achievable feed conversion ratio; Table S5: Overview of the predicted arginine doses needed to attain maximal, 99% and 95% of maximal achievable daily weight gain (DWG); Table S6: Overview of amino acid analysis of the starter diets. All values are expressed as g/100g of feed; Table S7: Overview of amino acid analysis of the grower diets. All values are expressed as g/100g of feed; Table S8: Overview of amino acid analysis of the finisher diets. All values are expressed as g/100g of feed.
