# Supplementary material for: An Evaluation of the Arginine Requirements of Broiler Chickens and the Potential Arginine and Energy-Saving Effects of Guanidinoacetic Acid
Source: Animals (Basel). 2024 Dec 24;15(1):4. doi: 10.3390/ani15010004 (PMC11718852; doi:10.3390/ani15010004)
Supplement: Supplementary file 1 [file animals-15-00004-s001.zip › animals-3320419-supplementary.pdf]

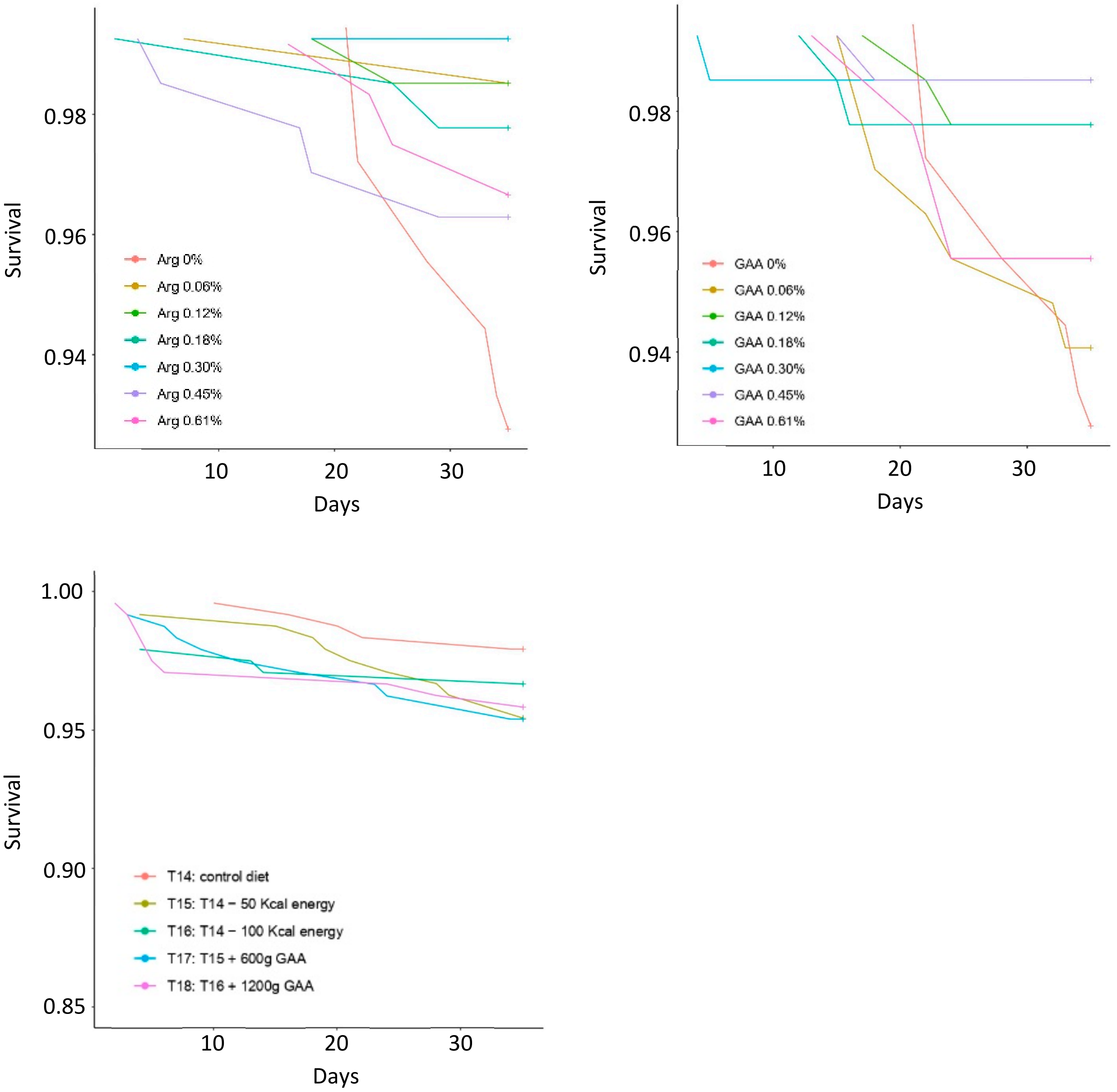

Supplementary Figure S1. Kaplan Meier survival graphs for the Arg (top left) and GAA (top right) supplementation groups (experiment 1), and the energy deficient diets + GAA (bottom) supplementation groups (experiment 2).

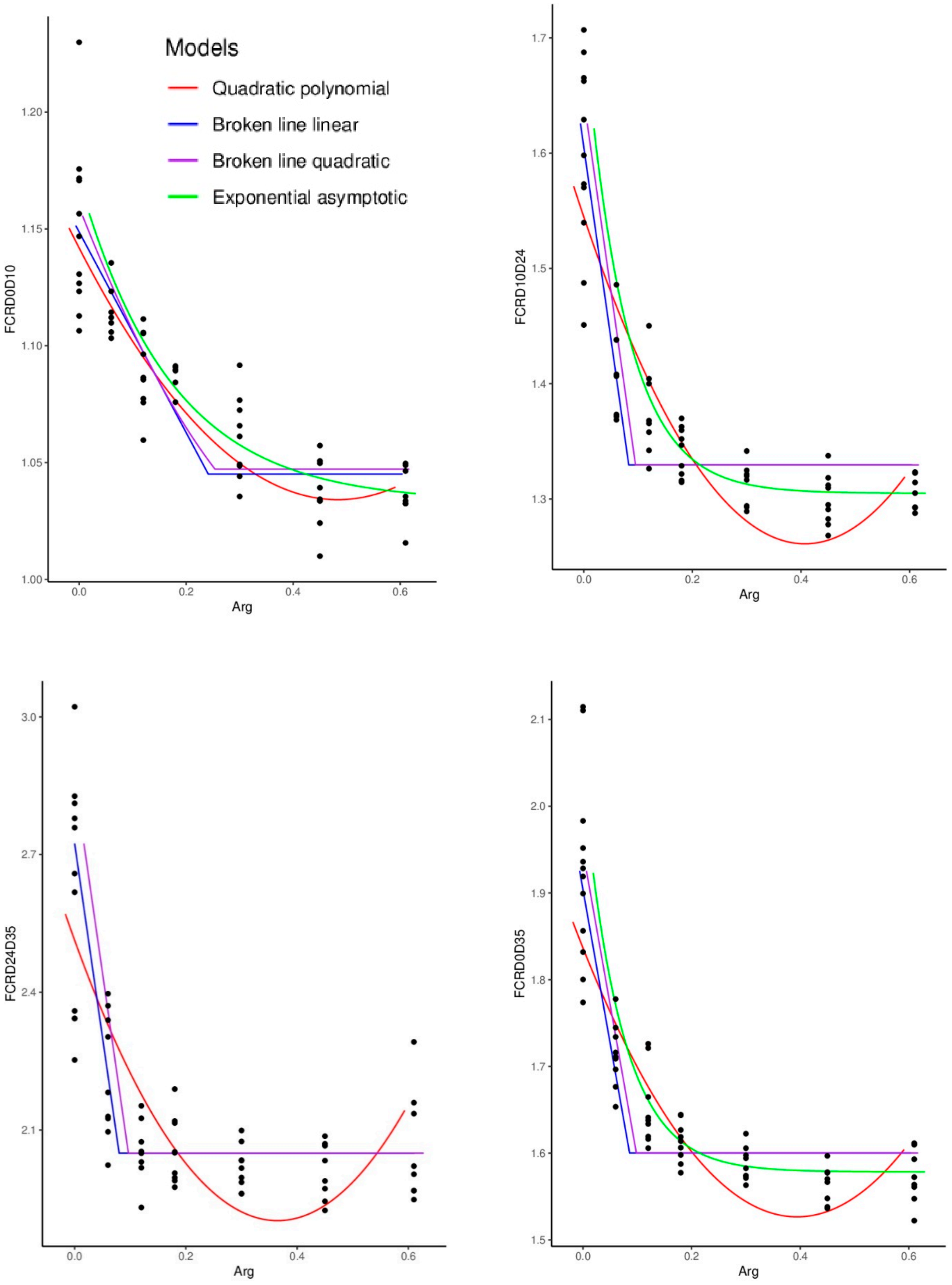

Supplementary Figure S2. Overview of the different fitted models for feed conversion ratio for Arg supplementation groups (experiment 1), during starter (top left), grower (top right) and finisher (bottom left) phase and overall (bottom right).

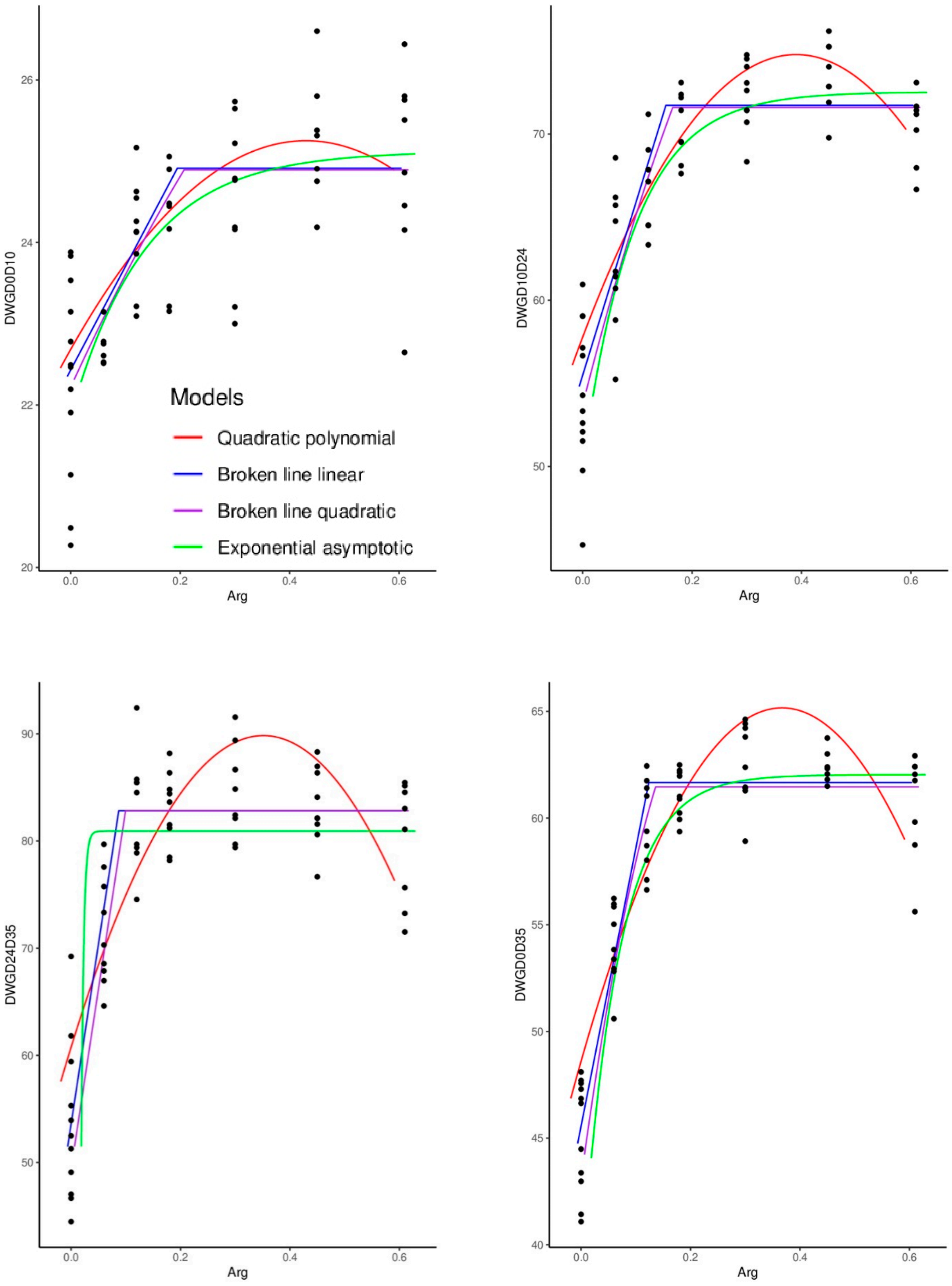

Supplementary Figure S3. Overview of the different fitted models for daily weight gain for Arg supplementation groups (experiment 1), during starter (top left), grower (top right) and finisher (bottom left) phase and overall (bottom right).

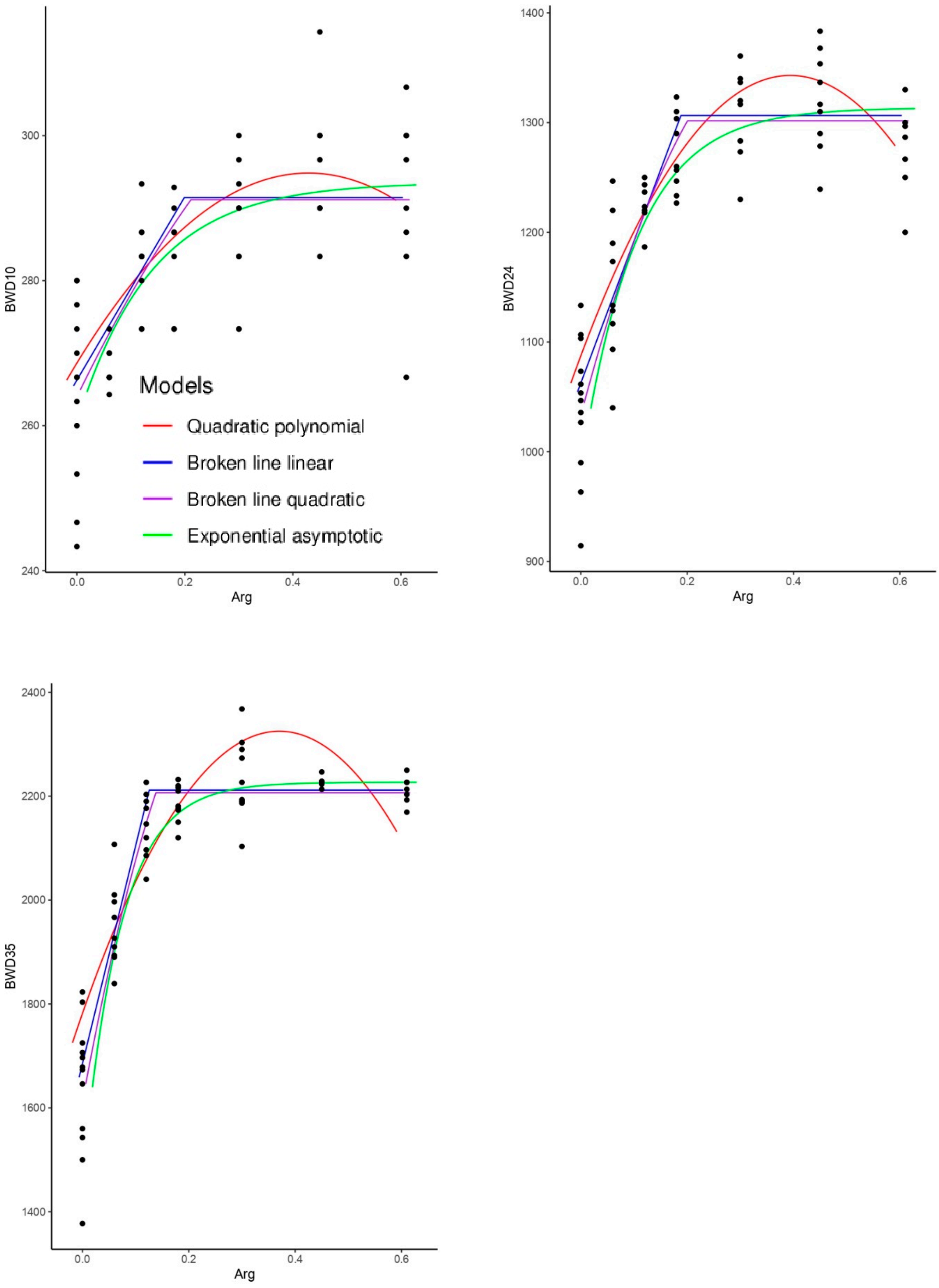

Supplementary Figure S4. Overview of the different fitted models for body weight for Arg supplementation groups (experiment 1), during starter (top left), grower (top right) and finisher (bottom left) phase.

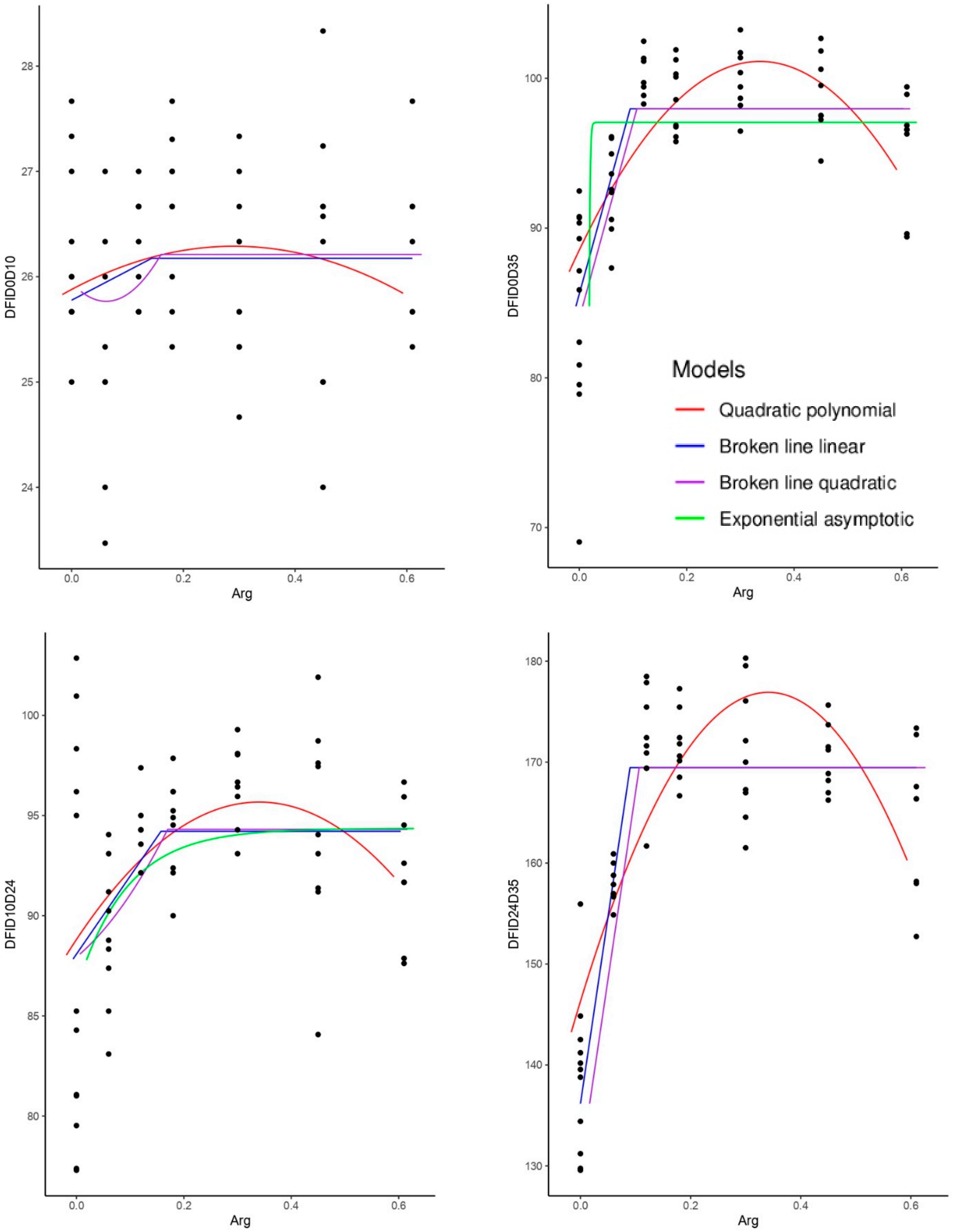

Supplementary Figure S5. Overview of the different fitted models for daily feed intake for Arg supplementation groups Supplementary Figure S4. Overview of the different fitted models for body weight for Arg supplementation groups (experiment 1), during starter (top left), grower (top right) and finisher (bottom left) phase and overall (bottom right).

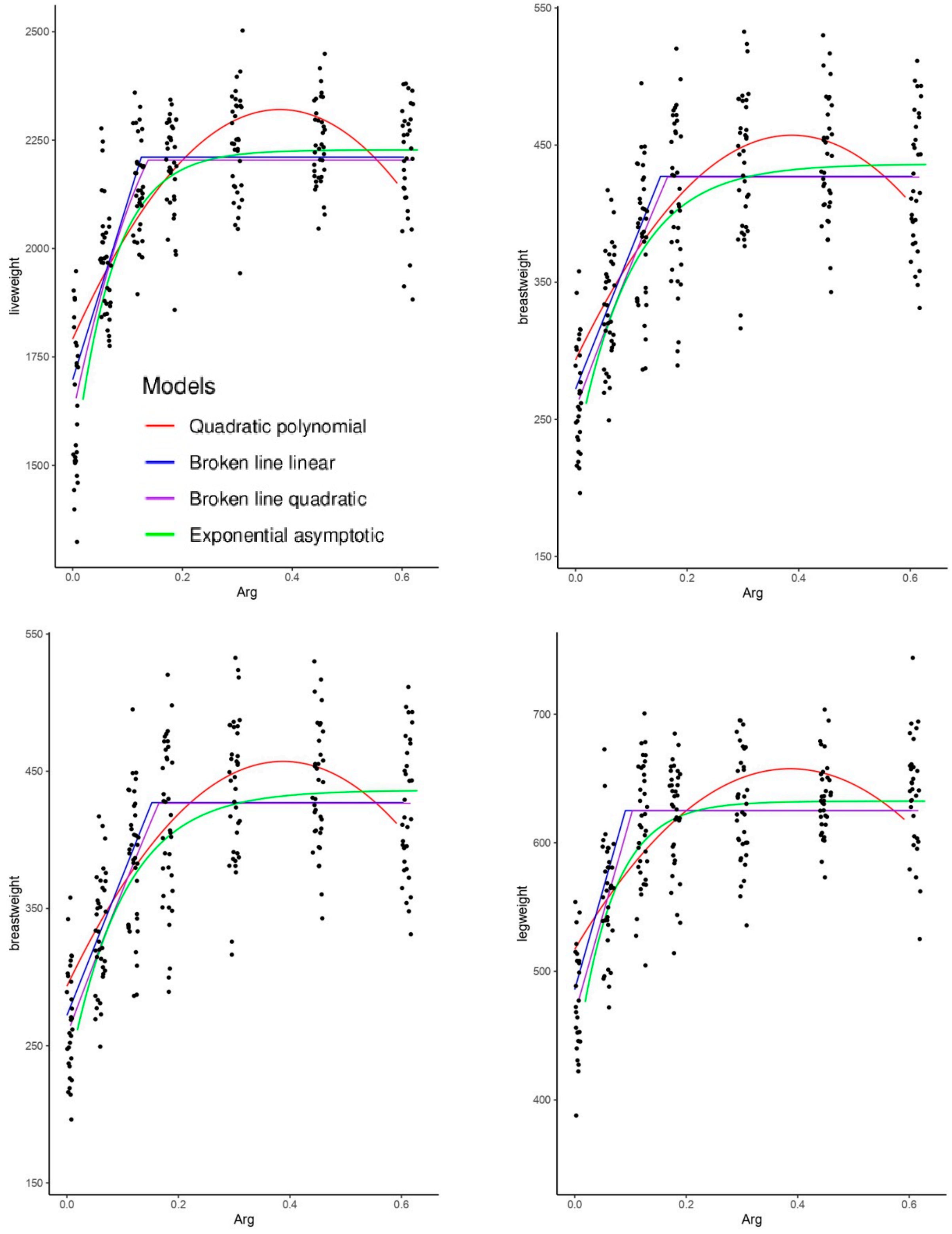

Supplementary Figure S6. Overview of the different fitted models for absolute slaughter parameters for Arg supplementation groups (experiment 1).

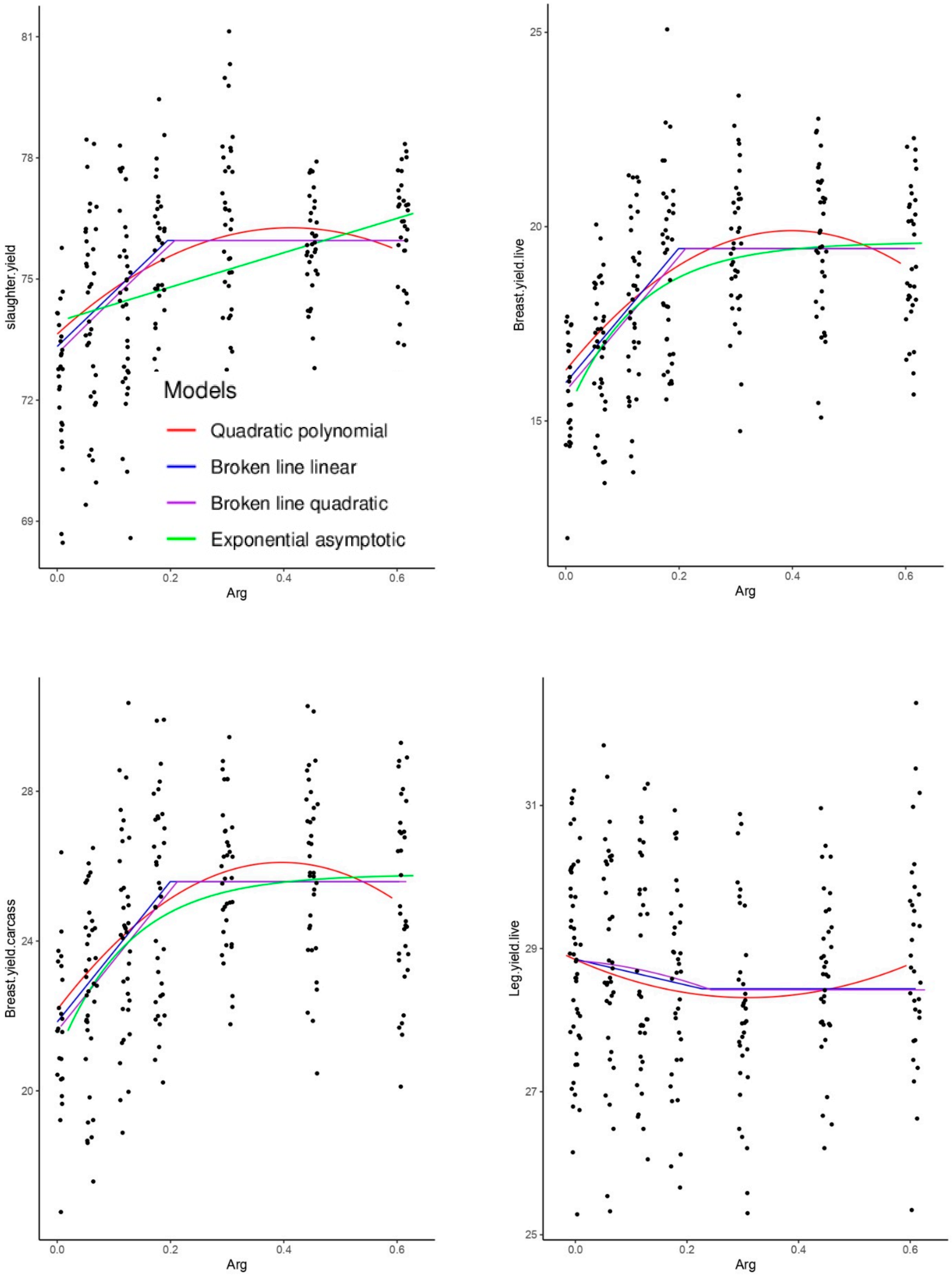

slaughter.yield
81
78
75
72
69
0.0
0.2
0.4
0.6
Arg
Models
Quadratic polynomial
Broken line linear
Broken line quadratic
Exponential asymptotic
Breast.yield.live
25
20
15
Breast.yield.carcass
28
24
20
Leg.yield.live
31
29
27
25

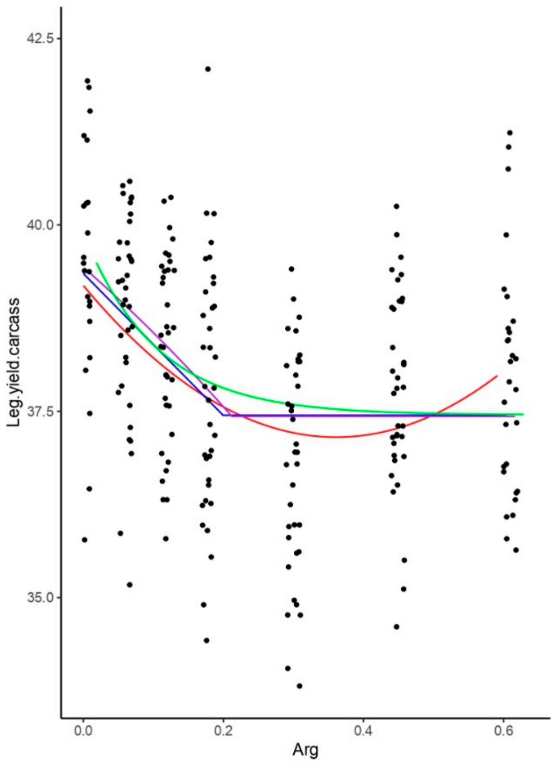

Supplementary Figure S7. Overview of the different fitted models for relative slaughter parameters for Arg supplementation groups (experiment 1).

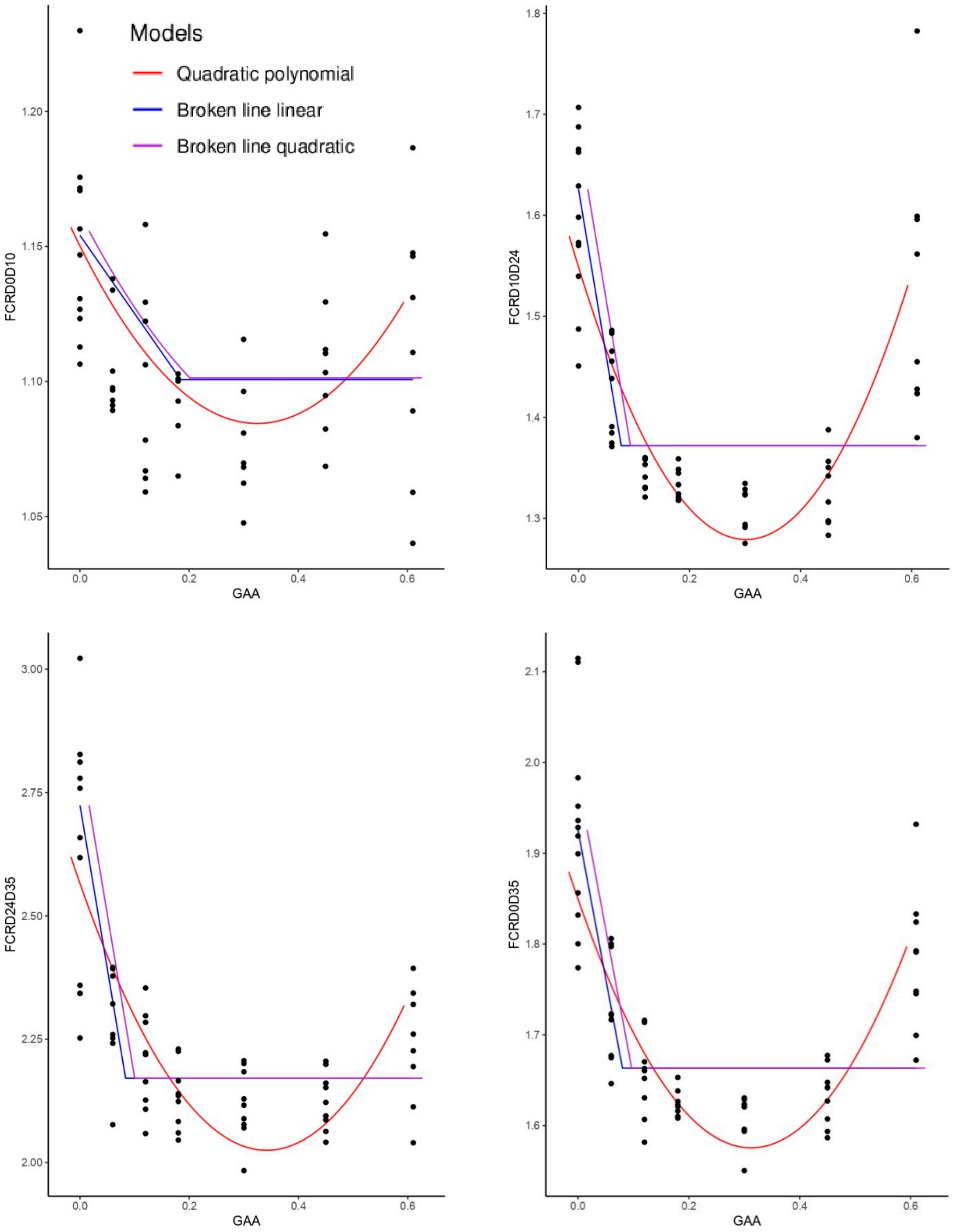

Supplementary Figure S8. Overview of the different fitted models for feed conversion for GAA supplementation groups (experiment 1), during starter (top left), grower (top right) and finisher (bottom left) phase and overall (bottom right).

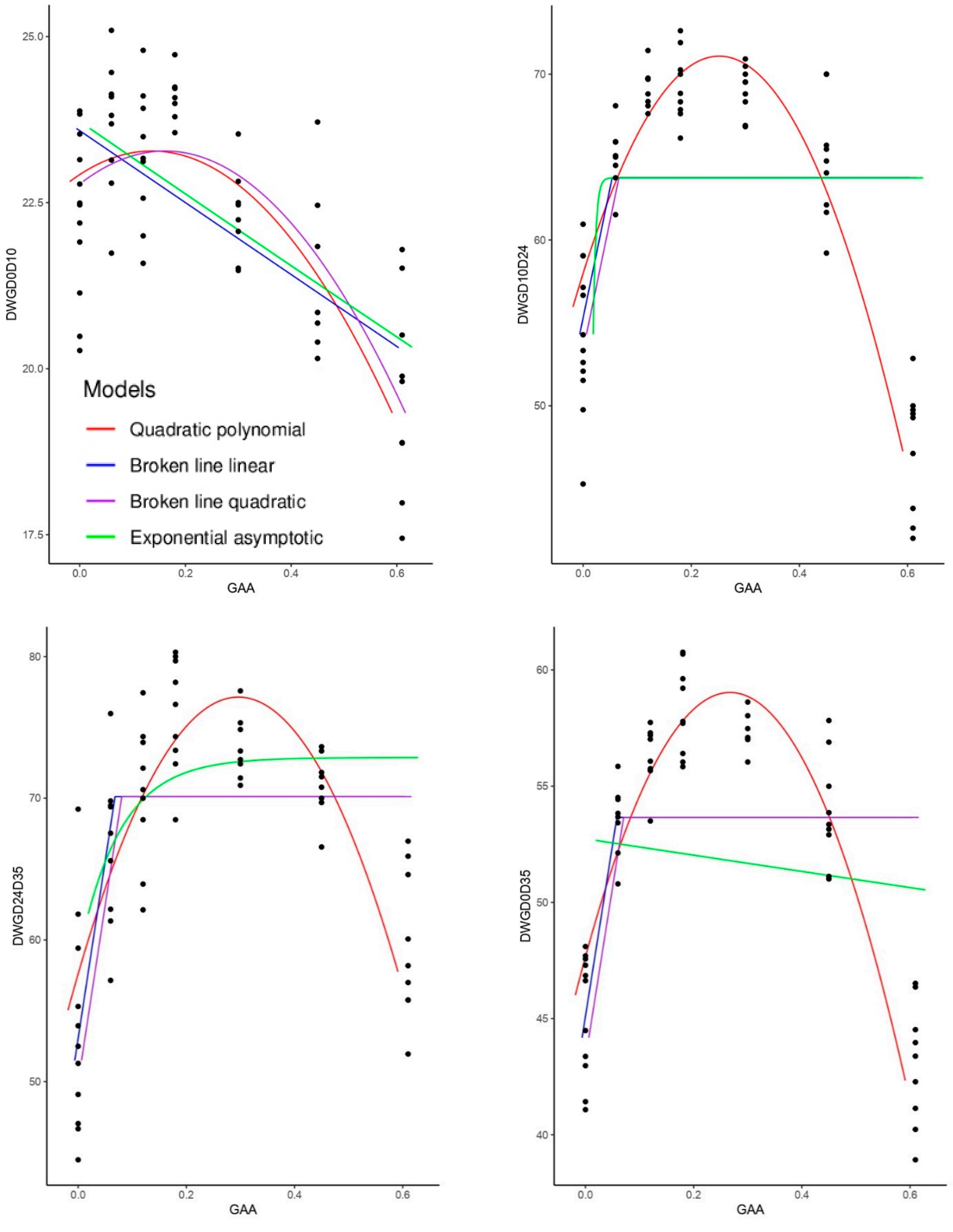

Supplementary Figure S9. Overview of the different fitted models for daily weight gain for GAA supplementation groups (experiment 1), during starter (top left), grower (top right) and finisher (bottom left) phase and overall (bottom right).

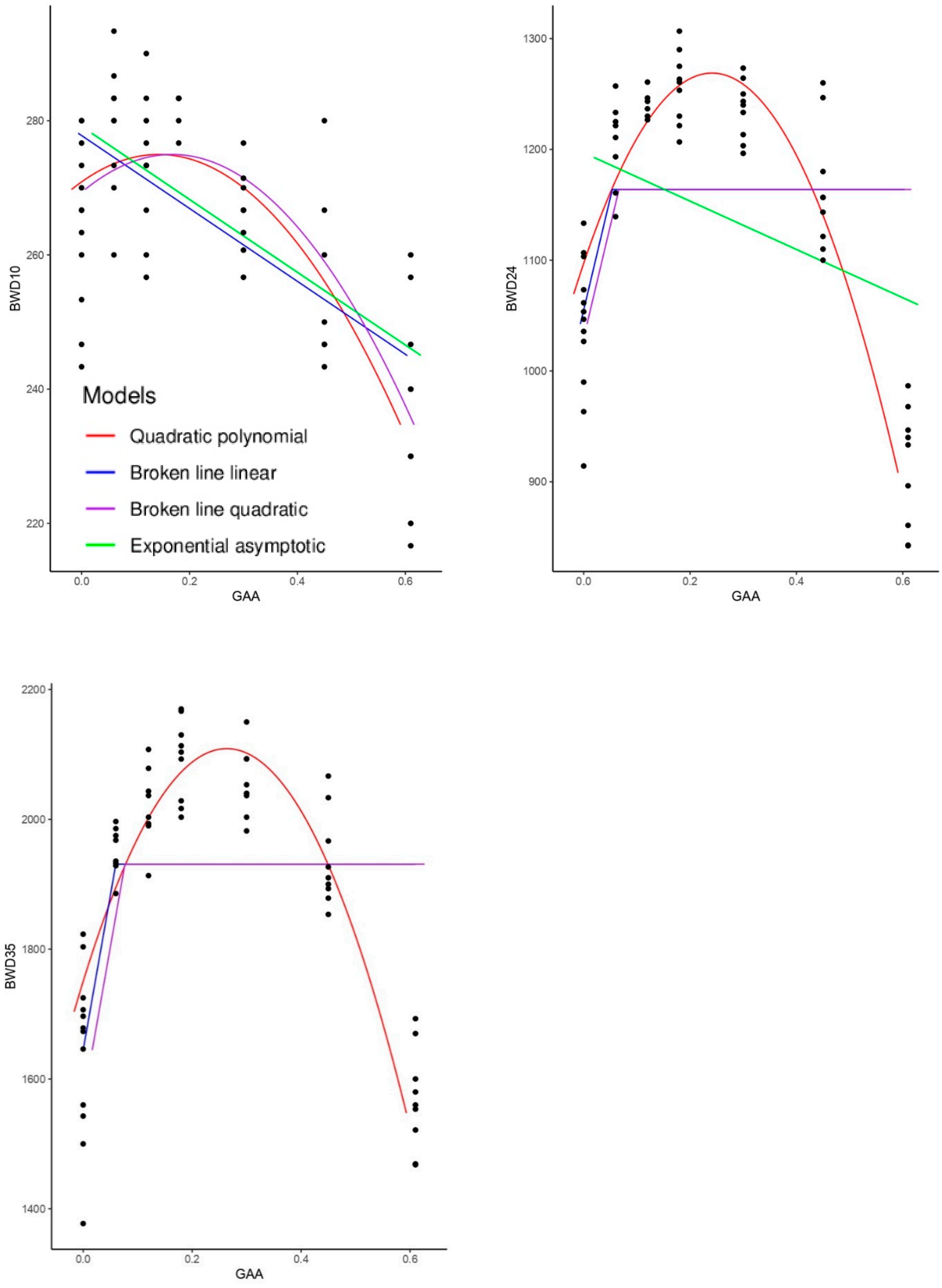

Supplementary Figure S10. Overview of the different fitted models for body weight for GAA supplementation groups (experiment 1), during starter (top left), grower (top right) and finisher (bottom left) phase.

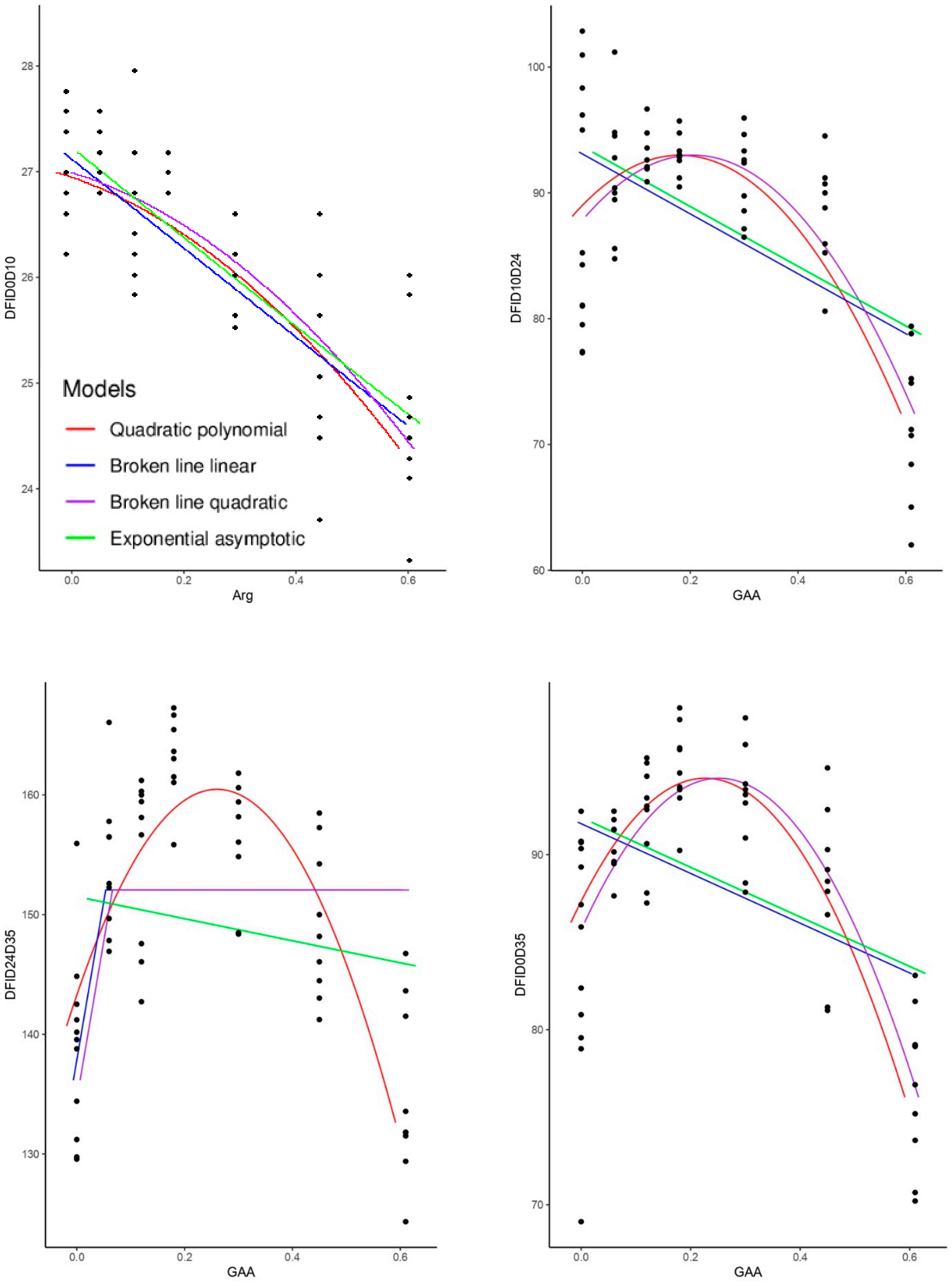

Supplementary Figure S11. Overview of the different fitted models for daily feed intake for GAA supplementation groups (experiment 1), during starter (top left), grower (top right) and finisher (bottom left) phase and overall (bottom right).

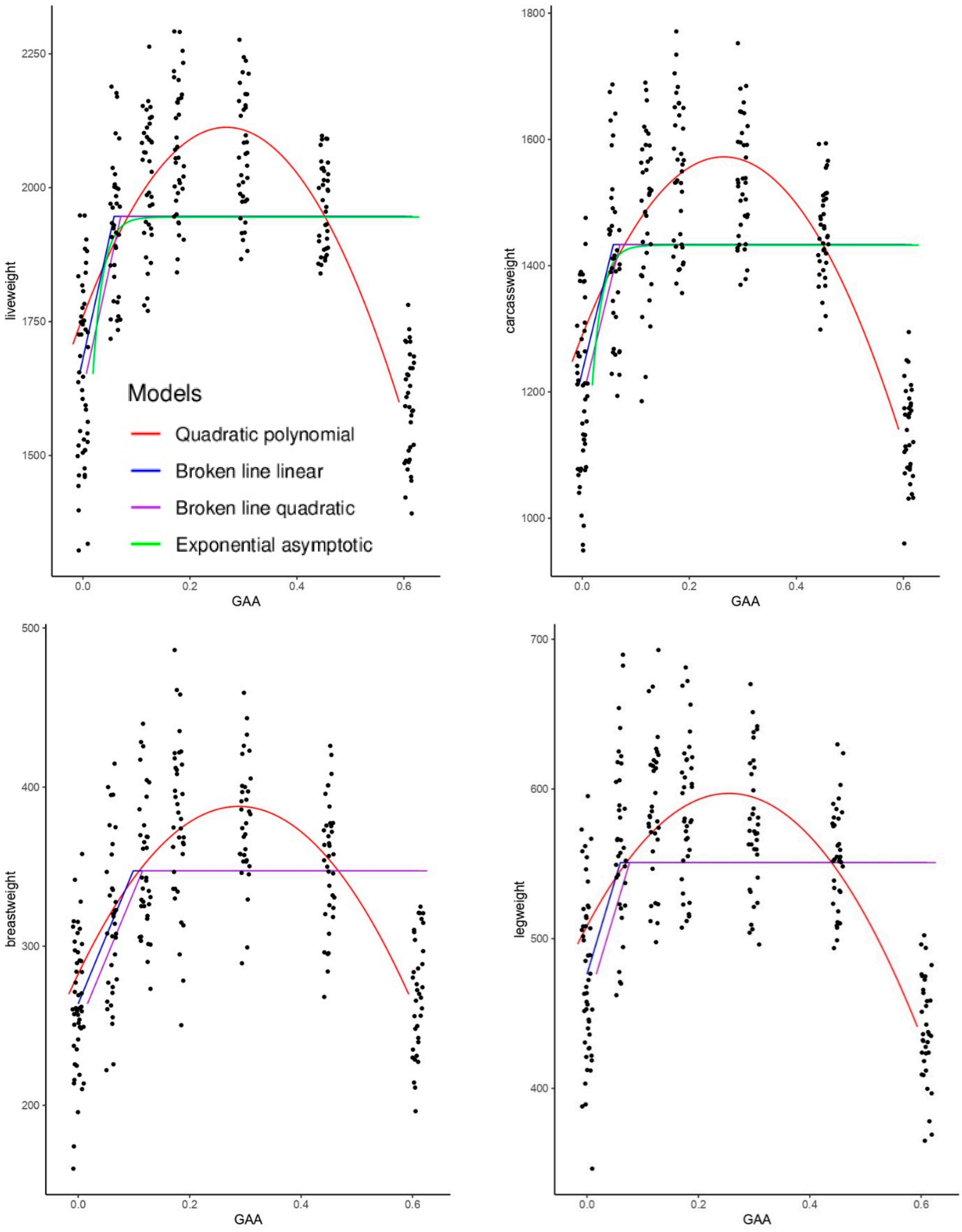

Supplementary Figure S12. Overview of the different fitted models for absolute slaughter parameters for GAA supplementation groups (experiment 1).

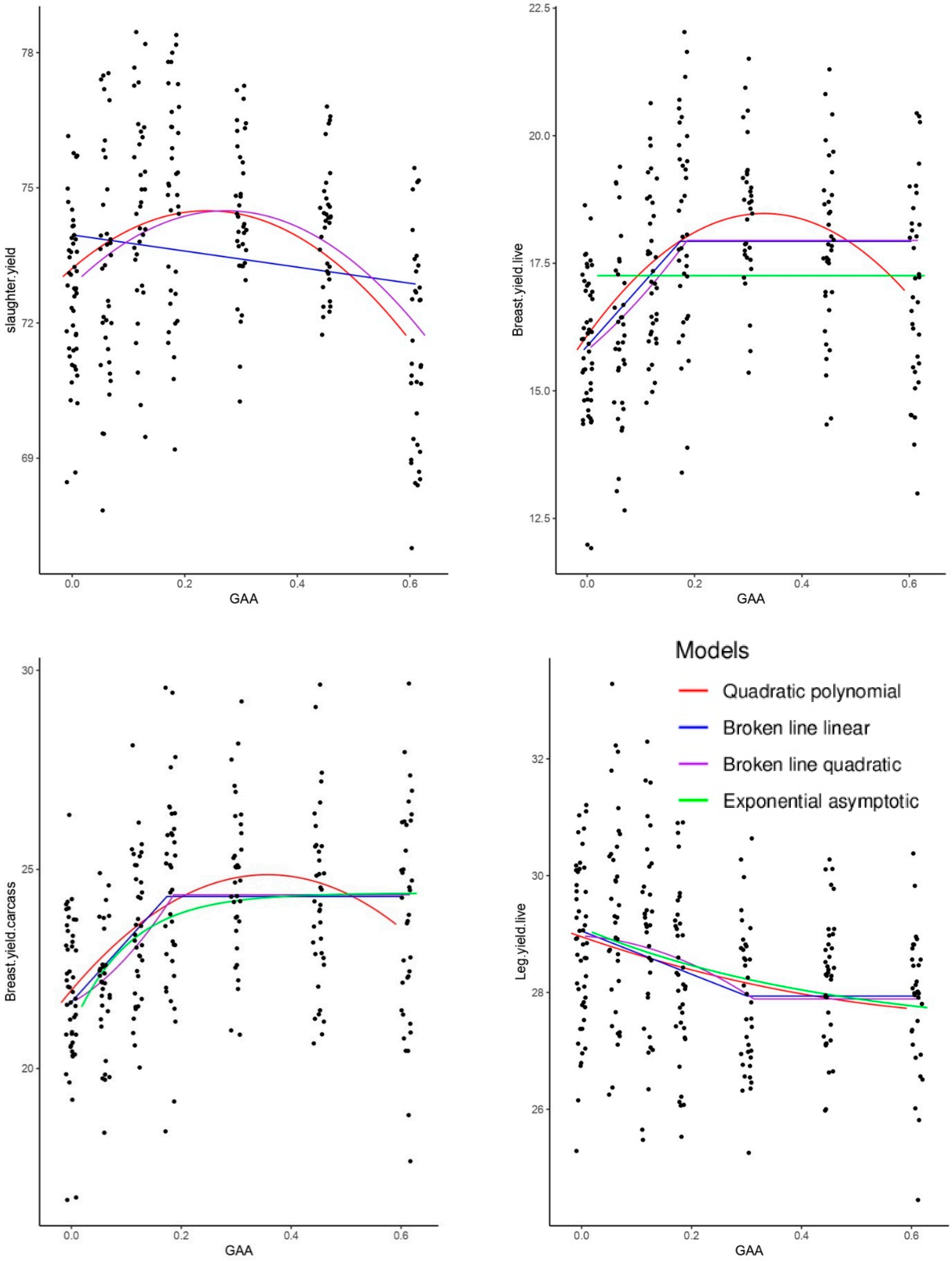

slaughter.yield
78
75
72
69
0.0
0.2
0.4
0.6
GAA
Breast.yield.live
22.5
20.0
17.5
15.0
12.5
Breast.yield.carcass
30
25
20
Leg.yield.live
32
30
28
26
Models
Quadratic polynomial
Broken line linear
Broken line quadratic
Exponential asymptotic

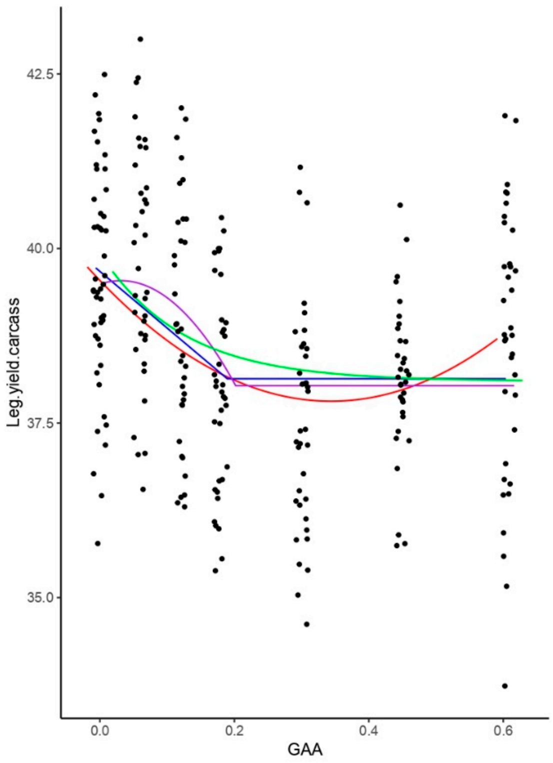

Supplementary Figure S13. Overview of the different fitted models for relative slaughter parameters for GAA supplementation groups (experiment 1).

Supplementary Table S1. Proximate and Gross Energy components of broiler starter

Treatment	Crude protein (%)	Crude fat (%)	Moisture (%)	Crude ash (%)	Crude fiber (%)	GE value (kcal/kg)
Experiment 1						
T01: Arg 0% (Basal diet)	21.6	5.2	11.55	5.06	2.3	
T02: Arg 0.06%	21.3	5.5	11.03	5.11	2.6	
T03: Arg 0.12%	21.9	5.6	11.29	5.24	2.7	
T04: Arg 0.18%	21.6	6.1	11.15	5.27	2.5	
T05: Arg 0.30%	21.6	5.6	11.37	5.15	2.5	
T06: Arg 0.45%	22.0	5.8	11.04	5.53	2.7	
T07: Arg 0.61%	22.4	5.5	11.05	5.39	2.8	
T09: GAA 0.12%	21.6	5.5	11.13	5.19	2.5	
T10: GAA 0.18%	21.4	5.6	11.00	5.24	2.6	
T11: GAA 0.30%	21.8	5.6	11.04	5.33	2.7	
T12: GAA 0.45%	22.3	5.5	11.06	5.22	2.7	
T13: GAA 0.61%	22.3	5.5	11.25	5.14	2.5	
Experiment 2						
T01: Control	22.7	5.2	12.26	5.38	2.2	3967
T02: T01 - 50 kcal	22.1	4.1	12.13	5.55	1.9	3910
T03: T01 - 100 kcal	22.1	3.3	12.10	5.61	2.3	3841
T04: T02 + 600g GAA	22.1	4.8	11.86	5.72	1.9	3891
T05: T03 + 1200g GAA	22.3	3.3	12.12	5.81	2.1	3812

GAA: guanidinoacetic acid; Arg: arginine

Supplementary Table S2. Proximate and Gross Energy components of broiler grower

Treatment	Crude protein (%)	Crude fat (%)	Moisture (%)	Crude ash (%)	Crude fiber (%)	GE value (kcal/kg)
Experiment 1						
T01: Arg 0% (Basal diet)	19.8	7.4	11.67	4.43	3.2	
T02: Arg 0.06%	19.6	8.4	11.49	4.54	2.9	
T03: Arg 0.12%	19.9	8.0	11.40	4.63	3.1	
T04: Arg 0.18%	20.0	8.2	11.33	4.62	2.9	
T05: Arg 0.30%	20.7	8.2	11.41	4.47	3.0	
T06: Arg 0.45%	21.2	7.9	11.58	4.42	2.9	
T07: Arg 0.61%	20.7	8.0	11.31	4.55	3.0	
T08: GAA 0.06%	19.7	8.2	11.41	4.38	2.9	
T09: GAA 0.12%	19.4	8.4	11.27	4.41	3.1	
T10: GAA 0.18%	20.6	8.8	11.54	4.32	2.9	
T11: GAA 0.30%	20.7	8.2	11.27	4.64	3.0	
T12: GAA 0.45%	20.2	7.9	11.51	4.29	2.9	
T13: GAA 0.61%	20.9	7.7	11.42	4.45	3.0	
Experiment 2						
T01: control	20.6	6.3	12.3	4.56	2.1	3996
T02: T01 - 50 kcal	20.2	5.5	12.36	4.79	2.1	3929
T03: T01 - 100 kcal	20.7	5.2	12.44	4.41	2.1	3855
T04: T02 + 600g GAA	20.9	5.7	12.42	4.67	1.9	3920
T05: T03 + 1200g GAA	20.0	5.5	12.60	4.69	1.9	3905

GAA: guanidinoacetic acid; Arg: arginine

Supplementary Table S3. Proximate and Gross Energy components of broiler finisher

Treatment	Crude protein (%)	Crude fat B (%)	Moisture (%)	Crude ash (%)	Crude fiber (%)	GE (kcal/kg)
Experiment 1						
T01: Arg 0% (Basal diet)	14.3	5.3	12.02	4.12	2.3	
T02: Arg 0.06%	14.7	5.5	12.06	3.98	2.1	
T03: Arg 0.12%	15.0	5.5	12.07	4.22	2.4	
T04: Arg 0.18%	15.3	5.4	11.92	4.15	3.1	
T05: Arg 0.30%	15.4	5.5	12.01	4.24	2.4	
T06: Arg 0.45%	15.8	5.5	11.85	4.18	2.7	
T07: Arg 0.61%	16.6	5.4	11.73	4.56	2.9	
T08: GAA 0.06%	15.4	5.4	12.1	4.19	2.5	
T09: GAA 0.12%	15.2	5.4	12.37	4.14	2.6	
T10: GAA 0.18%	15.2	5.6	12.02	4.19	2.4	
T11: GAA 0.30%	15.1	5.5	11.74	4.32	2.5	
T12: GAA 0.45%	15.2	5.6	11.82	4.36	2.5	
T13: GAA 0.61%	16.0	5.4	11.6	4.24	2.5	
Experiment 2						
T01: control	18.4	6.4	11.87	4.41	1.8	4018
T02: T01 - 50 kcal	18.2	5.6	12.25	4.38	1.9	3979
T03: T01 - 100 kcal	17.8	4.8	11.98	4.47	3.0	3865
T04: T02 + 600g GAA	18.3	5.6	12.26	4.29	2.4	3903
T05: T03 + 1200g GAA	18.7	5.0	12.19	4.34	2.0	3846

GAA: guanidinoacetic acid; Arg: arginine

Supplementary Table S4. Overview of the predicted arginine doses needed to attain maximal, 99% and 95% of maximal achievable feed conversion ratio.

**D0D10**	**AIC**	**Min FCR**	**Dose Min**	**101% FCR**	**Dose 101%**	**105% FCR**	**Dose 105%**
Quadratic polynomial	-279.7	1.034	0.50	1.044	0.35	1.086	0.17
Broken line linear	-275.0	1.045	0.25	1.056	0.22	1.097	0.13
Broken line quadratic	-274.0	1.047	0.25	1.058	0.21	1.100	0.11
Exponential asymptotic	-282.7	1.033	no fit	1.043	0.43	1.085	0.15
**D10D24**							
Quadratic polynomial	-144.0	1.261	0.43	1.274	0.34	1.324	0.23
Broken line linear	-155.5	1.330	0.09	1.343	0.08	1.396	0.07
Broken line quadratic	-153.5	1.330	0.09	1.343	0.08	1.396	0.07
Exponential asymptotic	-165.3	1.305	no fit	1.318	0.24	1.370	0.12
**D24D35**							
Quadratic polynomial	3.8	1.902	0.38	1.921	0.32	1.998	0.24
Broken line linear	-15.1	2.050	0.08	2.070	0.08	2.152	0.07
Broken line quadratic	-13.1	2.050	0.08	2.070	0.08	2.152	0.07
**D0D35**							
Quadratic polynomial	-154.6	1.527	0.41	1.542	0.33	1.603	0.22
Broken line linear	-178.4	1.600	0.09	1.616	0.09	1.680	0.07
Broken line quadratic	-176.4	1.600	0.09	1.616	0.09	1.680	0.07
Exponential asymptotic	-190.3	1.578	no fit	1.594	0.22	1.657	0.10

For each time interval, 4 different models and their corresponding Akaiki Information Criterion (AIC) are shown. For the finisher stage, D24-D35, an exponential asymptotic model could not be fitted. The most optimal model, based on the lowest AIC score, is underlined.

Supplementary Table S5. Overview of the predicted arginine doses needed to attain maximal, 99% and 95% of maximal achievable daily weight gain (DWG).

D0D10	AIC	Max DWG	Dose max	99% DWG	Dose 99%	95% DWG	Dose 95%
Quadratic polynomial	202.1	25.3	0.45	25.0	0.31	24.0	0.15
Broken line linear	201.6	24.9	0.20	24.7	0.18	23.7	0.10
Broken line quadratic	203.5	24.9	0.20	24.6	0.18	23.6	0.10
Exponential asymptotic	201.1	25.1	no fit	24.9	0.33	23.9	0.11
**D10D24**							
Quadratic polynomial	358.0	74.8	0.41	74.0	0.33	71.0	0.23
Broken line linear	349.4	71.7	0.16	71.0	0.15	68.1	0.12
Broken line quadratic	350.9	71.6	0.16	70.9	0.15	68.0	0.12
Exponential asymptotic	346.2	72.5	no fit	71.8	0.31	68.9	0.15
**D24D35**							
Quadratic polynomial	458.3	89.8	0.37	88.9	0.31	85.3	0.23
Broken line linear	424.4	82.8	0.09	82.0	0.09	78.7	0.08
Broken line quadratic	426.4	82.8	0.09	82.0	0.09	78.7	0.08
Exponential asymptotic	446.5	80.9	no fit	80.1	0.02	76.9	0.01
**D0D35**							
Quadratic polynomial	348.7	65.2	0.39	64.5	0.31	61.9	0.22
Broken line linear	316.6	61.7	0.13	61.0	0.13	58.6	0.11
Broken line quadratic	315.8	61.5	0.13	60.8	0.12	58.4	0.10
Exponential asymptotic	313.2	62.0	no fit	61.4	0.22	58.9	0.12

For each time interval, 4 different models and their corresponding Akaiki Information Criterion (AIC) are shown. The most optimal model, based on the lowest AIC score is underlined.

Supplementary Table S6. Overview of amino acid analysis of the starter diets. All values are expressed as g/100g of feed.

	T01 Arg 0%	T02 Arg 0.06%	T03 Arg 0.12%	T04 Arg 0.18%	T05 Arg 0.30%	T06 Arg 0.45%	T07 Arg 0.61%	T08: GAA 0.06%	T09: GAA 0.12%	T10 GAA 0.18%	T11 GAA 0.30%	T12 GAA 0.45%	T13 GAA 0.61%	T14	T15	T16	T17	T18
Lysine	1.41	1.43	1.41	1.37	1.45	1.43	1.37	1.45	1.48	1.39	1.44	1.5	1.46	1.43	1.42	1.43	1.47	1.43
Methionine, det. as Methionine sulfone, calc. as Methionine	0.64	0.68	0.63	0.59	0.65	0.68	0.62	0.66	0.68	0.64	0.66	0.67	0.64	0.6	0.61	0.62	0.64	0.69
Sum of Cystein and Cystin, det. as Cysteine acid, calc. as Cystine	0.37	0.37	0.36	0.33	0.36	0.35	0.38	0.37	0.37	0.38	0.36	0.38	0.35	0.42	0.36	0.36	0.38	0.35
Aspartic acid	1.8	1.82	1.79	1.76	1.79	1.77	1.76	1.8	1.76	1.79	1.79	1.79	1.78	2.16	2.11	2.17	2.22	2.13
Threonine	0.99	1.02	1	0.99	1.04	1.03	0.97	1.02	1.02	1	1.01	1.02	1.02	0.96	0.99	0.99	1.04	1.03
Serine	0.94	0.94	0.93	0.94	0.96	0.92	0.94	0.92	0.92	0.93	0.9	0.92	0.93	1.02	1.03	1.01	1.06	1.02
Glutamic acid	3.74	3.72	3.72	3.65	3.73	3.63	3.67	3.68	3.63	3.68	3.69	3.79	3.71	4.25	4.21	4.28	4.38	4.23
Proline	1.28	1.28	1.31	1.29	1.32	1.29	1.33	1.3	1.28	1.32	1.33	1.32	1.24	1.27	1.29	1.25	1.4	1.28
Glycine	1.06	1.08	1.06	1.04	1.1	1.09	1.05	1.11	1.13	1.06	1.11	1.13	1.11	0.9	0.88	0.88	0.92	0.88
Alanine	1.12	1.09	1.11	1.09	1.09	1.07	1.11	1.11	1.1	1.12	1.11	1.1	1.1	1.01	0.98	0.9	0.98	0.95
Valine	0.99	0.99	0.99	0.97	1.02	1.03	1.08	1.1	1.1	1.07	1.1	1.06	1.04	1.05	1.01	1.05	1.08	1.04
Isoleucine	0.96	0.97	0.95	0.91	0.96	0.93	0.96	0.99	0.99	0.94	1	0.99	0.97	0.98	0.97	0.95	0.96	0.93
Leucine	1.79	1.77	1.78	1.78	1.8	1.74	1.87	1.85	1.85	1.85	1.88	1.86	1.84	1.74	1.75	1.67	1.71	1.62
Tyrosine	0.72	0.75	0.67	0.67	0.73	0.69	0.69	0.68	0.66	0.65	0.7	0.7	0.69	0.73	0.71	0.66	0.68	0.69
Phenylalanine	1.06	1.06	0.93	0.93	1	0.99	0.96	0.98	0.97	0.98	1	0.99	0.97	1.06	1.03	1	1.05	1
Histidine	0.53	0.52	0.52	0.51	0.52	0.51	0.51	0.53	0.52	0.51	0.51	0.51	0.52	0.56	0.55	0.57	0.57	0.55
Arginine	1.2	1.24	1.29	1.31	1.48	1.58	1.66	1.17	1.16	1.09	1.16	1.24	1.17	1.54	1.5	1.51	1.47	1.48
Sum amino acids, calc.	20.6	20.73	20.45	20.13	21	20.73	20.93	20.72	20.62	20.4	20.75	20.97	20.54	21.68	21.4	21.3	22.01	21.3

Supplementary Table S7. Overview of amino acid analysis of the grower diets. All values are expressed as g/100g of feed.

	**T01 Arg 0%**	**T02 Arg 0.06%**	**T03 Arg 0.12%**	**T04 Arg 0.18%**	**T05 Arg 0.30%**	**T06 Arg 0.45%**	**T07 Arg 0.61%**	**T08: GAA 0.06%**	**T09: GAA 0.12%**	**T10 GAA 0.18%**	**T11 GAA 0.30%**	**T12 GAA 0.45%**	**T13 GAA 0.61%**	**T14**	**T15**	**T16**	**T17**	**T18**
Lysine	1.28	1.28	1.3	1.34	1.32	1.32	1.27	1.25	1.24	1.25	1.3	1.34	1.29	1.29	1.31	1.23	1.28	1.28
Methionine, det. as Methionine sulfone, calc. as Methionine	0.56	0.54	0.6	0.59	0.61	0.59	0.59	0.59	0.59	0.56	0.61	0.6	0.57	0.56	0.57	0.53	0.56	0.55
Sum of Cystein and Cystin, det. as Cysteine acid, calc. as Cystine	0.35	0.35	0.37	0.39	0.36	0.34	0.35	0.35	0.36	0.35	0.35	0.36	0.35	0.36	0.36	0.34	0.36	0.36
Aspartic acid	1.54	1.52	1.51	1.5	1.53	1.5	1.55	1.58	1.59	1.56	1.57	1.61	1.56	1.98	1.94	1.89	1.99	2
Threonine	0.9	0.91	0.93	0.94	0.92	0.93	0.92	0.92	0.93	0.89	0.94	0.99	0.9	0.88	0.91	0.86	0.9	0.9
Serine	0.84	0.82	0.86	0.83	0.85	0.83	0.86	0.88	0.89	0.83	0.86	0.9	0.82	0.93	0.98	0.93	0.99	0.98
Glutamic acid	3.48	3.45	3.53	3.46	3.5	3.44	3.43	3.47	3.45	3.43	3.45	3.47	3.49	4.14	4.08	3.95	4.1	4.09
Proline	1.39	1.28	1.28	1.26	1.26	1.23	1.2	1.25	1.23	1.3	1.19	1.27	1.18	1.28	1.3	1.3	1.31	1.34
Glycine	1.07	1.09	1.09	1.11	1.1	1.08	1.06	1.03	1.03	1.04	1.06	1.1	1.08	1.09	1.11	1.06	1.08	1.09
Alanine	1.08	1.06	1.07	1.05	1.06	1.04	1.02	1.03	1.07	1.09	1.08	1.1	1.09	0.89	0.95	0.93	0.95	0.94
Valine	0.94	0.94	0.94	0.96	0.97	0.94	0.91	0.93	0.92	0.93	0.94	0.95	0.96	0.97	0.96	0.96	0.97	0.99
Isoleucine	0.9	0.91	0.92	0.91	0.93	0.9	0.87	0.87	0.85	0.87	0.87	0.88	0.87	0.85	0.87	0.86	0.87	0.89
Leucine	1.77	1.79	1.77	1.73	1.78	1.75	1.68	1.7	1.72	1.71	1.69	1.7	1.74	1.58	1.62	1.61	1.62	1.64
Tyrosine	0.64	0.64	0.65	0.62	0.63	0.61	0.66	0.67	0.6	0.58	0.58	0.57	0.63	0.63	0.68	0.66	0.68	0.69
Phenylalanine	0.87	0.87	0.88	0.85	0.86	0.84	0.88	0.87	0.85	0.82	0.84	0.88	0.85	1	0.98	0.95	0.98	1.01
Histidine	0.5	0.5	0.5	0.49	0.5	0.49	0.5	0.5	0.49	0.49	0.49	0.51	0.49	0.54	0.52	0.52	0.52	0.53
Arginine	1.03	1.1	1.17	1.22	1.35	1.45	1.53	1.03	1.07	1.17	1.28	1.46	1.01	1.35	1.32	1.33	1.35	1.35
Sum amino acids, calc.	19.14	19.05	19.37	19.25	19.53	19.28	19.28	18.92	18.88	18.87	19.1	19.69	18.88	20.32	20.46	19.91	20.51	20.63

Supplementary Table S8. Overview of amino acid analysis of the finisher diets. All values are expressed as g/100g of feed.

	**T01 Arg 0%**	**T02 Arg 0.06%**	**T03 Arg 0.12%**	**T04 Arg 0.18%**	**T05 Arg 0.30%**	**T06 Arg 0.45%**	**T07 Arg 0.61%**	**T08: GAA 0.06%**	**T09: GAA 0.12%**	**T10 GAA 0.18%**	**T11 GAA 0.30%**	**T12 GAA 0.45%**	**T13 GAA 0.61%**	**T14**	**T15**	**T16**	**T17**	**T18**
Lysine	1.02	1.05	1.01	1.1	1.07	1.01	1.06	1.07	1.08	1.03	1.13	1.25	1.06	1.13	1.14	1.14	1.15	1.12
Methionine, det. as Methionine sulfone, calc. as Methionine	0.41	0.41	0.4	0.41	0.43	0.39	0.4	0.45	0.43	0.4	0.44	0.48	0.42	0.5	0.49	0.57	0.51	0.5
Sum of Cystein and Cystin, det. as Cysteine acid, calc. as Cystine	0.27	0.27	0.28	0.27	0.29	0.27	0.26	0.29	0.25	0.27	0.26	0.3	0.27	0.32	0.31	0.3	0.31	0.31
Aspartic acid	1.07	1.06	1.06	1.07	1.09	1.05	1.09	1.08	1.12	1.09	1.1	1.25	1.09	1.62	1.64	1.57	1.66	1.61
Threonine	0.64	0.66	0.65	0.68	0.69	0.62	0.68	0.67	0.7	0.68	0.72	0.81	0.69	0.79	0.8	0.82	0.81	0.79
Serine	0.6	0.56	0.59	0.59	0.6	0.55	0.6	0.57	0.62	0.62	0.62	0.68	0.6	0.81	0.85	0.81	0.85	0.83
Glutamic acid	2.38	2.35	2.37	2.37	2.39	2.38	2.38	2.37	2.46	2.43	2.4	2.73	2.44	3.71	3.69	3.61	3.7	3.64
Proline	1.03	0.98	0.94	0.97	0.99	0.95	1	0.96	0.92	0.93	1	0.95	0.91	1.08	1.21	1.18	1.19	1.16
Glycine	0.96	1.03	1	1.07	1.05	1.01	1.05	1.09	1.06	1	1.12	1.24	1.04	0.74	0.73	0.72	0.73	0.72
Alanine	0.79	0.79	0.79	0.79	0.79	0.8	0.8	0.79	0.78	0.78	0.78	0.86	0.77	0.82	0.83	0.8	0.82	0.82
Valine	0.7	0.71	0.71	0.72	0.72	0.71	0.72	0.72	0.71	0.69	0.7	0.79	0.69	0.89	0.88	0.88	0.89	0.87
Isoleucine	0.59	0.6	0.6	0.62	0.62	0.61	0.63	0.63	0.6	0.59	0.6	0.68	0.59	0.78	0.79	0.78	0.78	0.77
Leucine	1.21	1.17	1.19	1.18	1.21	1.21	1.21	1.2	1.18	1.19	1.14	1.25	1.17	1.38	1.43	1.38	1.4	1.39
Tyrosine	0.44	0.43	0.43	0.43	0.42	0.43	0.44	0.4	0.42	0.41	0.41	0.52	0.42	0.53	0.59	0.56	0.57	0.56
Phenylalanine	0.6	0.57	0.59	0.58	0.59	0.59	0.59	0.58	0.55	0.57	0.54	0.63	0.56	0.82	0.87	0.81	0.85	0.84
Histidine	0.38	0.38	0.38	0.38	0.38	0.38	0.38	0.37	0.39	0.39	0.39	0.44	0.39	0.46	0.47	0.46	0.47	0.47
Arginine	0.73	0.75	0.82	0.9	1.02	1.1	1.28	0.72	0.72	0.67	0.68	0.82	0.67	1.18	1.25	1.23	1.24	1.22
Sum amino acids, calc.	13.82	13.77	13.81	14.13	14.35	14.06	14.57	13.96	13.99	13.74	14.03	15.68	13.78	17.56	17.97	17.62	17.93	17.62
